# Assessments scales for the evaluation of health-related quality of life in Parkinson's disease, progressive supranuclear palsy, and multiple system atrophy: a systematic review

**DOI:** 10.3389/fpsyg.2024.1438830

**Published:** 2024-09-10

**Authors:** Maria Lucia Maiuolo, Roberto Giorgini, Maria Grazia Vaccaro, Alessio Facchin, Andrea Quattrone, Aldo Quattrone

**Affiliations:** ^1^Department of Medical and Surgical Sciences, Magna Graecia University of Catanzaro, Catanzaro, Italy; ^2^Neuroscience Research Centre, Magna Graecia University of Catanzaro, Catanzaro, Italy

**Keywords:** assessment tool, quality of life, systematic review, wellbeing, Parkinson's disease, progressive supranuclear palsy, multiple system atrophy, psychometrics

## Abstract

**Background:**

The concept of wellbeing is expansive and intricate, making it challenging to define precisely. Similarly, the instruments employed to assess wellbeing are complex and multifaceted. Therefore, it is more appropriate to refer to the notion of wellbeing as Health-Related Quality of Life (HRQoL), which is the central focus of many measures used to assess the feeling of wellbeing. This review aimed to identify the tools most commonly used to evaluate HRQoL in individuals with Parkinsonism—a group of movement disorders that negatively impact the quality of life due to the intricate interplay of symptoms, socio-demographic characteristics, and psychological factors. The main aim was to assess the psychometric properties of these measures in terms of validity and reliability.

**Methods:**

A literature review was conducted, focusing on research related to the assessment of HRQoL in connection to symptoms of Parkinsonism. This review included all studies that examined HRQoL using evaluation scales, exams, or self-reported questionnaires. The literature review was conducted using the databases Scopus and Web of Science and the search engine PubMed to identify studies published between 1996 and 2023. Only records that assessed HRQoL in individuals with Parkinson's disease and Parkinsonism were selected for evaluation.

**Results:**

A total of 393 records were examined, and eight tools were identified as the most frequently used in the evaluation of HRQoL.

**Discussion:**

The results show a significant gap in knowledge regarding the latent structure and measurement invariance of HRQoL measurements, which may have a significant influence on the interpretation of test outcomes. Moreover, there is a lack of clear divergent validity between HRQoL assessments and other tests used as predictors of HRQoL. This could represent a significant limitation, affecting the construct and criterion validity of HRQoL measures.

## 1 Introduction

Parkinsonism refers to a group of neurodegenerative disorders characterized by core mobility impairments resulting from pathological degeneration in specific brain regions. The most common form, Parkinson's disease (PD), affects 0.3% of the general population, with prevalence rates ranging from 1% to 5% in those aged 65 to 69 years and 1% to 3% in those aged 80 to 90 years (Arboleda-Montealegre et al., 2021; Simpson et al., 2021). The onset of symptoms in PD is associated with the loss of neurons in the nigrostriatal pathway, primarily due to an abnormal accumulation of Lewy bodies, which are complex agglomerates of proteins. The most prevalent motor symptoms (MS) include tremors, bradykinesia, stiffness, postural instability, musculoskeletal issues, gait impairment, motor fluctuations, and dyskinesia. These symptoms often lead to subsequent difficulties, such as an increased risk of falls (Kim et al., 2018; Josiah et al., 2012; Hechtner et al., 2014).

PD and other forms of Parkinsonisms are also characterized by non-motor symptoms (NMS), which include neuropsychiatric, sensory, autonomic, and sleep disorders. NMS can drastically impact patients' daily lives. For example, impairment of cognitive functioning and sensory perception can influence food consumption, leading to a lack of energy and weight loss (Akbar et al., 2015). Psychiatric comorbidities, such as anxiety and depression, are common in PD patients and worsen the prognosis, negatively affecting many aspects of their lives (D'Iorio et al., 2017; Chuquilín-Arista et al., 2021). MS and NMS vary in severity and form, depending on the specific type of Parkinsonism, and are generally associated with the partial absence of dopamine in extrapyramidal networks and reductions in white and gray matter in cortical and subcortical regions (Winter et al., 2011a,b).

Progressive supranuclear palsy (PSP) is another form of Parkinsonism, sharing symptoms with PD but also presenting distinct signs such as supranuclear vertical gaze palsy, which aids in differential diagnosis (Boxer et al., 2017, cited in Li et al., 2023). PSP is unresponsive to levodopa, resulting in a generally worse prognosis than PD due to the lack of effective therapy (Li et al., 2023). Individuals with PSP often experience substantial visual impairments combined with balance symptoms due to supranuclear center degeneration and cerebellar atrophy, respectively, which severely impact daily living and psychosocial functioning (Schrag et al., 2003). Comorbidities in PSP are related to the duration of the disease and include a broad spectrum of neuropsychiatric disorders (Schrag et al., 2006; Winter et al., 2010a,b).

Multiple system atrophy (MSA) is another rare and progressive neurodegenerative disorder within the Parkinsonism spectrum, characterized by autonomic dysfunction, cerebellar ataxia, and pyramidal symptoms (Schrag et al., 2006). MSA symptoms are linked to cortical and subcortical degeneration caused by the abnormal accumulation of Lewy bodies in nerve cells. Similar to PSP, MSA has a poor prognosis, with neuropsychiatric comorbidities often emerging as the disease progresses (Xiao et al., 2022). Individuals with MSA experience reduced psychosocial functioning due to impaired motor function and cognitive decline (Jecmenica-Lukic et al., 2018; Winter et al., 2010a,b; Du et al., 2018).

While life expectancy for individuals with PD is very close to that of the general population, MSA and PSP progress more rapidly. Currently, no therapies exist for Parkinsonism that can inhibit neurodegenerative processes, leading to the inevitable deterioration of function over time. The progression of MS and NMS often results in significant psychological consequences, which can compromise activities of daily living (ADLs), such as eating, cleaning, dressing, and working. The complex nature of network degradation associated with Parkinsonism can lead to psychiatric symptoms independent of MS and NMS severity (Simpson et al., 2021).

Parkinsonism presents a heterogeneous clinical manifestation, with symptoms that interact and collectively impact the quality of life (QoL) of those affected. QoL is a broad concept that often intersects with terms such as wellbeing and wellness. Due to this overlap, QoL is considered an umbrella term that encompasses both wellbeing and wellness (Benjamin and Looby, 1998). QoL includes several dimensions, such as spirituality, economic position, employment, interpersonal connections, and health (Benjamin and Looby, 1998). Particularly, researchers refer to “health-related quality of life” (HRQoL) to describe the ways in which perceptions of or direct repercussions from health status affect QoL (Lee et al., 2015; Global Parkinson's Disease Survey Steering Committee, 2002; Jenkins et al., 1990).

Numerous studies have documented how the complications of Parkinsonism impair HRQoL. Given the disparate clinical manifestations, scholars employ various designs to accurately measure impairment. A common approach involves quantifying symptom severity (e.g., through scale administration) as an independent variable to predict HRQoL measures. There is a well-established negative correlation between the severity of MS and HRQoL, as motor impairment directly affects ADLs and increases the risk of secondary injuries (Kim et al., 2018; Josiah et al., 2012; Hechtner et al., 2014).

Moreover, NMS appears to impact HRQoL negatively; for example, cognitive impairment, assessed through neuropsychological tests, is a significant predictor of HRQoL, **with** attentional deficits and decrease**d** executive functions lead**ing** to lower HRQoL (Leroi et al., 2011; Guo et al., 2015; Ou et al., 2017).

However, the impact of certain NMS, such as depression, anxiety, and autonomic symptoms, on HRQoL remains unclear, and whether these NMS can predict HRQoL is still questionable (Kadastik-Eerme et al., 2015; Schrag et al., 2006; Winter et al., 2010a,b; Kovács et al., 2016; Sanchez-Luengos et al., 2022; Bugalho et al., 2021; Gan et al., 2014; Li et al., 2010). One plausible reason for this discrepancy may lie in the operationalization of HRQoL, which involves the collection of techniques used to translate the construct of HRQoL and its subdomains into measurable variables. Consequently, it is crucial to analyze the validity and reliability of the measures used to estimate HRQoL in Parkinsonisms.

The construction of a tool for evaluating HRQoL in the Parkinsonism population frequently involves methods similar to those used in the validation of psychological tests. HRQoL assessments are typically self-reported and can be classified into generic and specific types, depending on whether the items address common factors impacting HRQoL or distinctive symptoms linked with a particular condition. This study aims to clarify the psychometric characteristics of the most frequently employed tests to measure HRQoL in PD, PSP, and MSA populations. While Lewy Body Dementia, Cortico-Basal Degeneration, and Frontotemporal Dementia are additional types of Parkinsonisms, as indicated by the study's findings, these conditions are not addressed (see the Section 3).

## 2 Method

### 2.1 Search strategy

The search was conducted using *PubMed, Scopus*, and *Web of Science* with a structured search strategy, focusing on scientific articles published from 1996 to 2023. The search was completed on 20 February 2023. The following search term**s** were used: ((parkinson) OR (parkinsonism) AND (english[Filter])) and (((“health-related quality of life”) AND ((questionnaire) OR (“self-report”) OR (scale))) AND (english[Filter])) on *Scopus* followed by an overall search query limited to articles in **E**nglish. A similar search strategy was then applied in PubMed and Web of Science using the terms ((parkinson) OR (parkinsonism)) and (((“health-related quality of life”) AND ((questionnaire) OR (“self-report”) OR (scale))).

### 2.2 Inclusion and exclusion criteria

The selection of research involving patients with Parkinson's disease (PD), progressive supranuclear palsy (PSP), and multiple system atrophy (MSA) for evaluating their HRQoL was conducted using the PICO (Patient, Intervention, Control, Outcome) criteria (Higgins and Green, 2011; Table 1). Studies were conducted if they lacked a power analysis or had a sample size of fewer than 50 participants. Additionally, records that did not align with the objectives of the systematic review were excluded. Due to the lack of HRQoL data on other types of parkinsonism within the research approach used in this systematic review, only PD, PSP, and MSA were considered in this analysis.

**Table 1:** PICO.

Population of interest	Patients with PD and Parkinsonism
Intervention of interest	Use of measurement's instruments to evaluate Health-Related Quality of Life in PD and Parkinsonism
Comparison interventions	Not applicable
Outcomes	HRQoL in Parkinson's disease and Parkinsonism
Time	From 1996 to 2023
Other considerations	Sample size < 50 and no power analysis carried out

### 2.3 Investigated psychometric properties

Construct validity was assessed by examining the latent structure of the tests, analyzing the number of latent variables, and determining how the observed variables were associated with specific or general factors to gauge the level of agreement between the recorded findings. All articles that investigated the underlying structure of HRQoL measures were included, regardless of the specific statistical analysis methods used, such as confirmatory factor analysis (CFA) or exploratory factor analysis (EFA).

To evaluate the robustness of tests' latent structure, the literature review included all articles that analyzed measurement invariance (ME/I; Gregorich, 2006). In order to guarantee that test results remain consistent across different groups, testing ME/I is essential (Gregorich, 2006). For example, several studies have reported differences in HRQoL between men and women (Ophey et al., 2018). Another reason for including ME/I studies is that many of the measurements have undergone cross-cultural validation. This validation is necessary to guarantee that a test is appropriately adapted to different cultural contexts, making ME/I studies crucial.

While a clear and replicable latent structure is necessary, it is not sufficient to fully establish the construct validity of a test. Therefore, all paradigms of convergent and divergent validity for HRQoL measure were included, with particular attention given to divergent validity due to the theoretical overlap of constructs related to QoL. The Cronbach's alpha and other reliability indices of each test were also reported. When considering the usage of Cronbach's alpha, it is important to focus on the assumption of tau-equivalence in the latent structure. Significant emphasis was placed on this assumption check, as it is essential for ensuring unbiased results when using Cronbach's alpha (Flake et al., 2017).

The criterion validity of HRQoL tests was reported by assessing the main predictors of HRQoL, as well as by measuring comorbidities (e.g., depression) and MS and NMS scales. This review specifically focused on the rationale behind these predictions rather than investigating the predictor measures' psychometric properties. Additionally, validation techniques that followed the Item Response Theory framework were also included in the analysis.

## 3 Results

### 3.1 Identification of records

**Figure 1:**
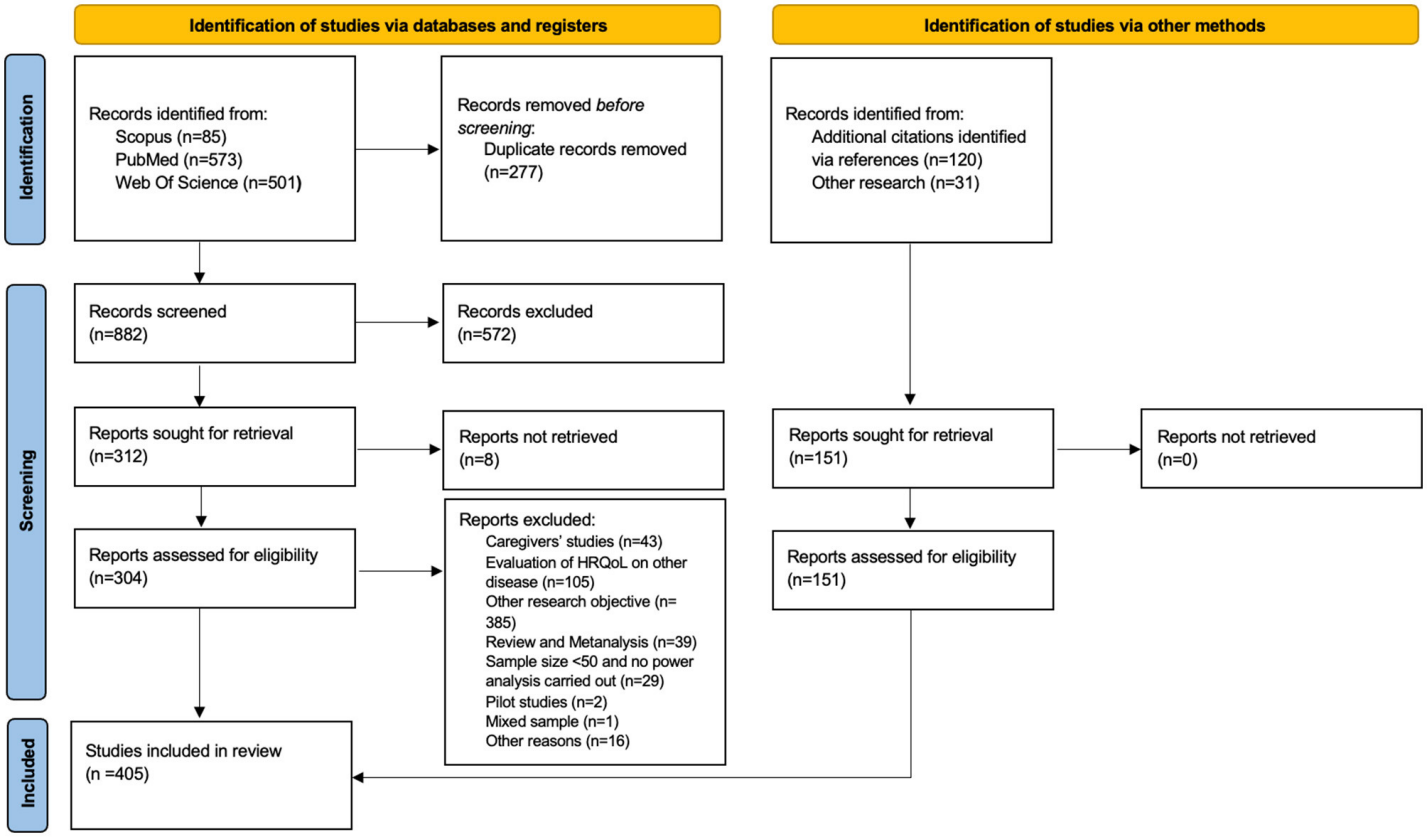
PRISMA flow diagram. PRISMA diagram for the selection of studies.

The PRISMA (Preferred Reporting Items for Systematic Review and Meta-Analyses) diagram (Page et al., 2021a,b) summarizes the search results, article screening process, and exclusions. A total of 1,159 records were identified from the search engines and databases, comprising 85 from Scopus, 573 from PubMed, and 501 from Web of Science (Figure 1). After the initial screening, 572 records and 277 duplicates were excluded (Figure 1). Further exclusions were made for studies focused on caregivers (*n* = 43), HRQoL in other diseases (*n* = 105), irrelevant research aims (*n* = 385), and systematic reviews (*n* = 39).

During the second screening process, additional exclusions were made for non-retrievable studies (*n* = 8), studies not aligned with our research objective or identified as pilot studies (*n* = 2), studies with mixed samples (*n* = 1), and those with a small sample size and lacking a clearly reported power analysis (*n* = 29). Moreover, 16 studies were excluded due to the complete absence of a method description or unclear methodology. An additional 151 records were identified through references or other research efforts. Finally, a total of 405 studies were included in this systematic review.

### 3.2 Identification of measures

The total number of HRQoL assessment tools and other tests applied in specific study designs was *n* = 121 (see Table 2).

**Table 2:** List of the instruments found for measuring HRQoL and predictors.

	**Physical**	**Mental**	**Social**	**ADL**	**Overall**	**Other**	**References**
15D generic instruments	✓	✓	✓	✓	-	-	Sintonen, xbib1994
Activities-Specific Balance Confidence Scale	-	-		✓	-	-	Powell and Myers, 1995
ADL (Activities of Daily Living)	-	-	-	✓	-	-	Lawton and Brody, 1969
AIS (Athens Insomnia Scale)	✓	-	-	-	-	-	Soldatos et al., 2000
Apathy Scales	-	✓	-	-	-	-	Starkstein et al., 1992
BAI (Beck Anxiety Inventory)	-	-	-	-	-	✓	Beck et al., 1993
Barthel Index of ADL	-	-	-	✓	-	-	Mahoney and Barthel, 1965
BBS (Berg Balance Scale)	-	-	-	-	-	✓	Berg et al., 1995
BDI (Beck Depression Inventory)	-	-	-	-	-	✓	Beck et al., 1987
Behave-AD (Behavior Pathology in Alzheimer's Disease Rating Scale)	-	-	-	-	-	✓	Reisberg et al., 1987
BELA-P-k (Belastungsfragebogen Parkinson's kurzversion)	✓	✓	✓	-	-	-	Ringendahl et al., 2000
BFAS (Big Five Aspects Scale)	-	-	-	-	-	✓	DeYoung et al., 2007
BIS-11 (Barratt Impulsiveness Scale)	-	-	-	-	-	✓	Patton et al., 1995
CESD (Center for Epidemiologic Studies Depression Scale)	-	-	-	-	-	✓	Radloff, 1977
CGI (Clinical Global Impression of Change)	-	-	-	-	-	✓	Weitkunat et al., 1993
CISI-PD (Clinical Impression Of Severity Index-Parkinson's Disease)	✓	✓	-	✓	-	✓	Martínez-Martín et al., 2003
COMPASS (Composite Autonomic symptom scale)	-	-	-	-	-	✓	Suarez et al., 1999
Composite International Diagnostic Interview Short Form for Major Depression	-	-	-	-	-	✓	Kessler et al., 1998
CISS (Coping Inventory for Stressful Situations)	-	-	-	-	-	✓	Endler and Parker, 1999
CSQ (Coping Strategies Questionnaire)	-	-	-	-	-	✓	Rosenstiel and Keefe, 1983
DASS-21 (Depression Anxiety Stress Scales)	-	-	-	-	-	✓	Lovibond and Lovibond, 1995
ESES (Exercise Self-Efficacy Scale)	-	-	-	-	-	✓	Kroll et al., 2007
ESS (Epworth Sleepness Scale)	-	-	-	-	-	✓	Johns, 1991
PDCS (European Parkinson's Disease Association Sponsored)	-	-	✓	✓	-	✓	Stocchi et al., 2018
EUROQoL	✓	✓	✓	✓	✓	-	Euroqol Group, 1990
FACIT (Functional Assessment of Chronic Illness Therapy)	✓	✓	✓	✓	✓	✓	Webster et al., 1999
FBI (The 24-item Frontal Behavioral Inventory)	-	-	-	-	-	✓	Kertesz et al., 1997
FES (Falls Efficacy Scale)	-	✓	-	✓	-	-	Tinetti et al., 1990
FFRT (Forward Functional Reaching Test)	-	-	-	-	-	✓	Duncan et al., 2014
FKV-LIS-SE (the Freiburg Coping with Disease Questionnaire)	-	-	-	-	-	✓	Muthny, 1989
FOG-Q (Freezing of Gait Questionnaire)	✓	-	-	-	✓	-	Giladi et al., 2000
FSI (Fatigue Severity Inventory)	-	-	-	-	-	✓	Lee et al., 1991
FSQ (Functional Status Questionnaire)	-	-	-	-	-	✓	Jette et al., 1986
FSS (Fatigue Severity Scale)	✓	-	-	✓	-	-	Krupp et al., 1989
GDS-15 (Geriatric Depression Scale)	-	-	-	-	-	✓	Yesavage et al., 1982
GSE (General Self-Efficacy Scale)	-	-	-	-	-	✓	Schwarzer and Jerusalem, 1999
German Essen Coping Questionnaire	-	-	-	-	-	✓	Franke et al., 2000
HandY stage (Hoehn and Yahr stage)	-	-	-	-	-	✓	Hoehn and Yahr, 1967
HADS (Hospital Anxiety and Depression Scale)	-	-	-	-	-	✓	Zigmond and Snaith, 1983
HAM-A (Hamilton Anxiety Rating Scale)	-	-	-	-	-	✓	Hamilton, 1959
HAM-D (Hamilton Depression Rating Scale)	-	-	-	-	-	✓	Hamilton, 1960
HUI-3 (Health Utilities Index Mark)	-	-	-	-	✓	-	Furlong et al., 1998
HWS (Holistic Wellbeing Scale)	✓	✓	-	-	✓	✓	Chan et al., 2014
IADL (Instrumental Activities of Daily Living)	-	-	-	✓	-	-	Lawton and Brody, 1969
Impulsive-Compulsive Disorders in Parkinson's Disease	-	-	-	-	-	✓	Weintraub et al., 2009
IPAQ (International Physical activity Questionnaire)	-	-	-	✓	-	✓	Cardol et al., 2001
IQCODE ('e Informant Questionnaire on Cognitive Decline in the Elderly)	-	-	-	-	-	✓	Cherbuin and Francis Jorm, 2010
King's Parkinson's Disease Pain Scale	✓	-	-	-	-	✓	Chaudhuri et al., 2015
LARS (Lille Apathy Rating Scale)	-	✓	-	-	-	-	Sockeel et al., 2006
Leed Anxiety and Depression scale	-	-	-	-	-	✓	Snaith et al., 1976
Livingston's Insomnia Scale	-	-	-	-	-	✓	Livingston et al., 1993
LOT-R (Life Orientation Test Revised)	-	-	-	-	-	✓	Scheier and Carver, 1985
MAAS (Mindful Attention Awareness Scale)	-	-	-	-	-	✓	Brown and Ryan, 2003
MADRS (Montgomery and Asberg Depression Rating Scale)	-	-	-	-	-	✓	Neumann and Schulte, 1989
MSS (Marital Satisfaction Scale)	-	-	-	-	-	✓	Roach et al., 1981
MDRS (Modified Dyskinesia Rating Scale)	✓	-	-	-	-	-	Goetz et al., 1994
MDS-UPDRS (Movement Disorder Society- Unified Parkinson's Disease Rating Scale)	-	-	-	-	-	✓	Goetz et al., 2008
Mini-BESTest (The Mini-Balance Evaluation Systems Test)	–	-	-	-	-	✓	Horak et al., 2009
MAS-QoL(MSA health-related Quality of Life scale)	✓	✓	✓	-	-	-	Schrag et al., 2007
MSPQ (Modified Somatic Perception Questionnaire)	-	-	-	-	-	✓	Main, 1983
NAS (Nottingham Adjustment Scale)	-	-	-	-	-	✓	Dodds et al., 1991
NEURO-QOL (Quality of Life in Neurological Disorders)	✓	✓	✓	✓	✓	-	Gershon et al., 2012
NHP (Nottingham Health Profile)	✓	✓	✓	✓	✓	-	Hunt et al., 1985
NMS-Quest (Non-Motor Symptoms Questionnaire)	✓	✓	-	-	-	✓	Romenets et al., 2012
NMSS (Non-Motor Symptom Scale)	-	✓	-	-	-	✓	Chaudhuri et al., 2007
OARS (The Older Americans Resources and Services)	✓	✓	✓	✓	✓	-	Fillenbaum and Smyer, 1981
PAS (Parkinson's Anxiety Scale)	-	-	-	-	-	✓	Leentjens et al., 2014
PANAS (The Positive and Negative Affect Schedule)	-	✓	-	-	-	-	Watson et al., 1988
PCIG (Patient Global Impression of Change)	-	-	-	-	-	✓	Ferguson and Scheman, 2009
PCQ-PD (Patient-Centered Questionnaire for PD)	-	-	-	-	-	✓	van der Eijk et al., 2012
PDQ-39 (Parkinson's Disease Questionnaire-39 item)	✓	✓	✓	✓	✓	✓	Peto et al., 1995
PDQ-8 (Parkinson's Disease Questionnaire-8 item)	✓	✓	✓	✓	✓	✓	Jenkinson et al., 1997
PDQL (Parkinson's disease quality of life questionnaire)	✓	✓	✓	-	✓	✓	de Boer et al., 1996
PDQualif (the Parkinson's Disease Quality of Life Scale)	-	✓	✓	✓	-	✓	Welsh et al., 2003
PDSS (Parkinson's disease sleep scale)	-	-	-	-	-	✓	Chaudhuri et al., 2002
PWI-A (Personal Wellbeing Index-Adult)	✓	✓	✓	✓	✓	✓	Lau et al., 2005
PFS-16 (Parkinson's Fatigue Scale)	-	-	-	-	-	✓	Brown et al., 2005
PGIC (Additional secondary measures of Patient Global Impression of Change)	✓	✓	-	-	✓	-	Hurst and Bolton, 2004
PHQ-9 (Patient Health Questionnaire)	-	-	-	-	-	✓	Kroenke et al., 2001
PILL Questionnaire (Impact of Cognitive Dysfunction on Daily Living Activities)	-	✓	-	✓	-	-	Dubois et al., 2007
PROMIS (The patient-reported outcomes measurement information system)	✓	✓	✓	-	✓	-	Ader, 2007
PSP-QoL (Progressive Supranuclear Palsy Rating Scale)	✓	✓	✓	✓	-	-	Schrag et al., 2006
PSP-RS (Progressive Supranuclear Palsy Rating Scale)	✓	-	-	-	-	✓	Golbe and Ohman-Strickland, 2007
PSQI (Pittsburgh Sleep Quality Index)	-	-	-	✓	-	✓	Buysse et al., 1989
PWS (Psychological Wellbeing Scale)	-	-	-	-	✓	✓	Ryff, 1989
QOL-AD (QOL Alzheimer's Disease)	-	-	-	✓	-	✓	Logsdon et al., 1999
QUEST (Quality of Life in Essential Tremor Questionnaire)	✓	✓	✓	✓	✓	✓	Tröster et al., 2005
RAD (Rapid Assessment of Disability Scale)	-	-	✓	✓	✓	-	Martinez-Martin et al., 2005
RBDSQ (REM Sleep Behaviour Disorder Symptoms Questionnaire)	-	-	-	-	-	✓	Stiasny-Kolster et al., 2007
RCSQ (Richards–Campbell Sleep Questionnaire)	-	-	-	-	-	✓	Richards, 1987
RSE (Rosenberg Self-Esteem Scale)	-	-	-	-	-	✓	Rosenberg, 1965
Ryff's scale of Psychological Wellbeing	-	-	-	-	✓	✓	Ryff, 1989; Ryff and Keyes, 1995
SandE (Schwab and England scale)	-	-	-	✓	-	-	Schwab and England, 1969
SAMS (German Stendal Adherence with Medication Score)	-	-	-	-	-	✓	Franke and Jagla-Franke, 2020
SCOPA-PS (Scale for Outcomes in Parkinson's Disease -Psychosocial questionnaire)	-	✓	✓	-	-	-	Marinus et al., 2003
SDS (Self-rating Depression Scale)	-	-	-	-	-	✓	Zung, 1965; Biggs et al., 1978
SEE (Self-Efficacy for Exercise scale)	-	-	-	-	-	✓	Resnick and Jenkins, 2000
SCOPA-AUT (Self-reported Autonomic Symptoms in Parkinson's Disease)	-	-	-	-	-	✓	Visser et al., 2004
SF-12 (Short-Form Health Survey 12 item)	✓	✓	✓	✓	✓	-	Ware et al., 1996
SF-36 (Short-Form Health Survey 36 item)	✓	✓	✓	✓	✓	-	Ware and Sherbourne, 1992
SF-6D (Short-Form Six Dimension)	✓	✓	✓	✓	✓	-	Brazier et al., 2002
Short Social Support Questionnaire	-	-	✓	-	-	-	Jahanshahi and Marsden, 1988; Sarason et al., 1983
SIP (Sickness Impact Profile)	✓	-	✓	✓	-	-	Bergner et al., 1976
SIPA (Social influences on physical Activity questionnaire)	-	-	-	-	-	✓	Chogahara, 1999
SOC-29 (Sense of Coherence Scale)	-	-	-	-	-	✓	Antonovsky, 1972
SOFAS (Social and occupational functioning assessment scale)	-	-	✓	✓	-	✓	Rybarczyk, 2011
SPMSQ (The Short Portable Mental Status Questionnaire)	✓	✓	✓	✓	-	✓	Pfeiffer, 1975
STAI (State Trait Anxiety Inventory)	-	-	-	-	-	✓	Spielberger et al., 1970
UMSARS (Unified Multiple System Atrophy Rating Scale)	✓	-	-	✓	-	✓	Wenning et al., 2004
UPDRS (Unified Parkinson's Disorder Rating Scale)	-	-	–	-	-	✓	Fahn, 1987
WCQ (Ways of Coping Questionnaire)	-	-	-	-	-	✓	Folkman and Lazarus, 1985
WHO-5 (World Health Organization Well Being Index 5 item)	-	-	-	-	-	✓	World Health Organization, 1999
WHO-DAS (World Health Organization -Disability Assessment Schedule)	-	-	✓	✓	-	✓	Ustün et al., 2010
WHO-DAS II (World Health Organization -Disability Assessment Schedule-II)	✓	-	✓	✓	✓	✓	World Health Organization, 1999
WHOQOL-100 (The World Health Organization Quality of Life)	✓	✓	✓	✓	✓	✓	The WHOQOL Group, 1998; Whoqol Group, 1998
WPAI-GH (The Work Productivity and Activity Impairment Questionnaire-General Health)	-	-	-	-	-	✓	Reilly et al., 1993
AES (Apathy Evaluation Scale)	-	-	-	-	-	✓	Marin et al., 1991; Santangelo et al., 2014
ZUF-8 (Patient Satisfaction Questionnaire)	-	-	-	-	-	✓	Schmidt et al., 1989
Starkestain's Apathy Scale (Structured Clinical Interview for Apathy)	-	-	-	-	-	✓	Starkstein et al., 2001
Zung-Depression Inventory-Self Rating Depression Scale	-	-	-	-	-	✓	Zung, 1965, 1972

The main instruments found in more than 3 records include 25 tools, with the most commonly used being:

- Specific instruments for evaluating HRQoL in Parkinsonisms: PDQ-39, PDQ-8, PDQL, and SCOPA-PS.
- Generic instruments for evaluating HRQoL: SF-36, SF-12, EuroQol-5 (EQ-5D-5L and EQ-5D-3L), and NHP.
- Instruments for evaluating predictors of HRQoL outcomes in PD, PSP, and MSA: MDS-UPDRS, CISI-PD, H&Y Stage, S&E, UMSARS, NMS-Quest, and NMSS.

**Figure 2:**
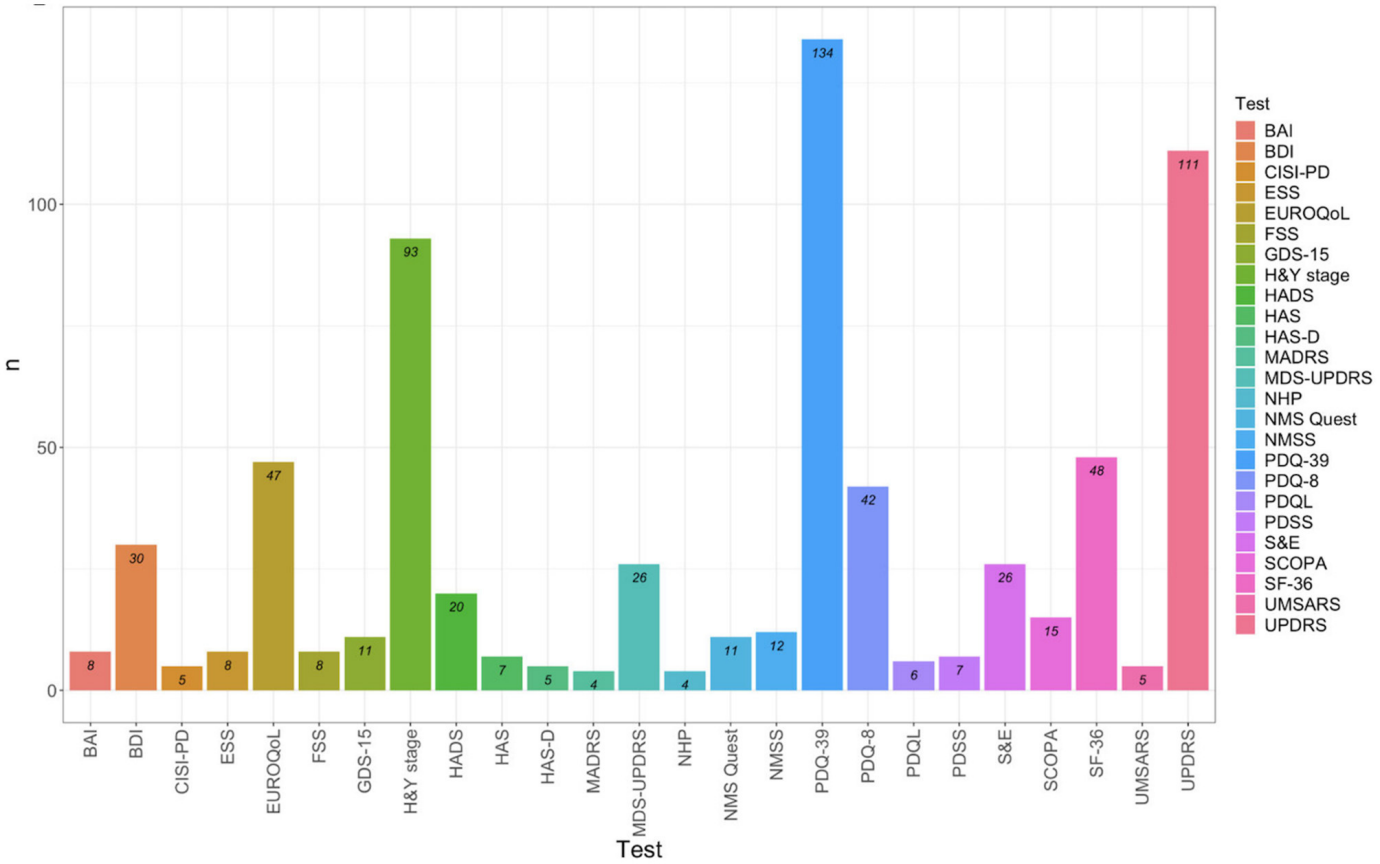
Test identified in more than 3 records. BAI, Beck Anxiety Inventory; BDI, Beck Depression Inventory; CISI-PD, Clinical Impression of Severity Index for Parkinson's Disease; ESS, Empworth Sleepiness Scale; EUROQoL, EuroQol group's test; FSS, Fatigue Severity Scale; GDS-15, Geriatric Depression Scale; HandY stage, Hoehn and Yahr stage; HADS, Hospital Anxiety and Depression Scale; HAM-A, Hamilton Anxiety rating scale; HAM-D, Hamilton Rating scale for depression; MADRS, Montgomery-Asberg Depression Rating Scale; MDS-UPDRS, Movement Disorder Society-Sponsored Revision of the Unified Parkinson's Disease Rating Scale; NHP, Nottingham Health Profile; NMS-Quest, Non-Motor Symptoms Questionnaire; NMSS, Non-motor symptoms scale; PDQ-39, Parkinson's Disease Questionnaire 39 items; PDQ-8, Parkinson's Disease Questionnaire 8 items; PDQL, Parkinson's Disease Quality of Life Questionnaire; PDSS, Parkinson's Disease SleepScale 2^nd^ version; SandE: Schwab and England Scale; SCOPA, Scales for Outcomes in Parkinson's Disease-Psychosocial Functioning; SF-36, 36 and 12 items Short-Form Health Survey; UMSARS, Unified Multiple System Atrophy Rating Scales; UPDRS, Unified Parkinson's Disease Rating Scale.

These instruments are illustrated in Figure 2.

### 3.3 Specific instruments to evaluate HRQoL in PD, PSP, and MSA

#### 3.3.1 PDQ-39

The 39-item Parkinson's Disease Questionnaire (PDQ-39) is one of the most widely used instruments for evaluating HRQoL in individuals with Parkinson's disease (Peto et al., 1995). It comprises 39 items that are divided into eight scales: mobility (eight items), Activities of Daily Living (ADL; six items), emotional wellbeing (six items), stigma (four items), social support (three items), cognitions (four items), communication (three items), and bodily discomfort (three items). Each item is scored on a scale from 0 to 4, and the total score, which is called the PDQ-39 Summary Index (PDQ-39SI), ranges from 0 to 100. The PDQ-39 is available in 14 languages: English, Spanish, American, Greek, Chinese, Singaporean, Ecuadorian, French, Brazilian, Iranian, Portuguese, Korean, Estonian, and Italian (Galeoto et al., 2018). It is also used in populations with MSA and PSP (Schrag et al., 2006).

Several studies have assessed the reliability of the PDQ-39, with generally acceptable values reported. However, most applications of Cronbach's alpha did not account for tau-equivalence, which could affect the accuracy of the reliability estimates. The construct validity of the PDQ-39 appears to be somewhat clear. Some reports indicate that some items may load on different scales than originally intended, raising questions about the clarity of the questionnaire's structure (Schönenberg et al., 2023). Furthermore, the latent structure of the PDQ-39 has been evaluated in only a few studies, which shows that the different latent variables are strongly related (Schönenberg et al., 2023). Moreover, while several studies have reported gender differences in PDQ-39 scores (e.g., Ophey et al., 2018), the ME/I of the PDQ-39 scores has not been thoroughly examined. Furthermore, no studies were found that assessed ME/I of the PDQ-39 across different countries.

#### 3.3.2 PDQ-8

The 8-item Parkinson's Disease Questionnaire (PD1-8) consists of eight items derived from PDQ-39 using Principal Component Analysis (PCA), with item selection based on item-factor correlations (Jenkinson et al., 1997). The total score, known as PDQ-8 SI, ranges from 0 to 100 (Luo et al., 2009). The PDQ-8 has been reported to be reliable (Chen et al., 2017; Martínez-Martín et al., 2003; Alvarado-Bolaños et al., 2015; Tan et al., 2004; Li et al., 2023; Franchignoni et al., 2008), but Cronbach's alpha varies across studies, typically ranging between 0.72 and 0.8.

The rationale for creating a short form of the PDQ-39 originates from the results of the hierarchical principal component analysis conducted by Jenkinson et al. (1997). This analysis extracted one component, suggesting that the eight dimensions of the PDQ-39 reflect a high-order factor. Furthermore, these results support the interpretation of item sum scores in both the PDQ-39 and PDQ-8 as general indices of HRQoL. However, results from Rasch analysis suggest that the PDQ-8 may not effectively differentiate HRQoL as a continuum and that its structure is not unidimensional (Franchignoni et al., 2008). Similar to the PDQ-39, no records were found that checked for tau-equivalence despite widespread reporting of Cronbach's alpha. Additionally, no studies were identified that tested for ME/I.

#### 3.3.3 PDQL

The Parkinson's Disease Quality of Life Questionnaire (PDQL) was developed by de Boer et al. (1996) and consists of 37 items divided into four dimensions: *Parkinsonian symptoms, systemic symptoms, emotional functioning*, and *social functioning*. The total score is calculated by summing the scores of each dimension, with a higher score indicating better HRQoL. The four dimensions of the PDQL were identified through EFA, and the reliability was evaluated by testing the internal consistency of subscales using Cronbach's alpha (de Boer et al., 1996).

Moreover, the convergent validity of the PDQL was tested by examining the correlation between its subscales and the Medical Outcome Studies-24 (MOS-24), a generic wellness test. In the initial validation and subsequent studies, PDQL demonstrated good reliability, with Cronbach's alpha exceeding 0.80 for each subscale and 0.90 for the total score (de Boer et al., 1996). However, despite this high internal consistency, the correlation between the PDQL and MOS-24 subscales was weak, particularly for the “Social Functioning” dimension, where the correlation ranged from 0.13 to 0.43.

Moreover, the factor structure of the PDQL includes complex loadings, meaning that some items appear to reflect more than one dimension simultaneously. No confirmatory studies were found to validate the PDQL's structure or justify the sum score. Furthermore, cross-cultural validation studies did not include ME/I across different countries or genders.

#### 3.3.4 SCOPA-PS

The Scales for Outcomes in Parkinson's Disease-Psychosocial (SCOPA-PS) is a self-report tool composed of 11 items on a 0–3 Likert scale (Marinus et al., 2003). The outcome of the SCOPA-PS is summarized in a summary index (SI), where a higher score indicates poorer HRQoL. The content of items reflects various social scenarios of daily living in which patients may have experienced suffering or difficulty in the previous month. Since its initial validation, SCOPA-PS has demonstrated good convergent validity through correlations with PDQ-39 and other generic tests. The factor structure appears unidimensional, although some indices, such as the RMSEA, are mediocre (RMSEA > 0.08). Reliability is generally good (Soulas et al., 2016; Virués-Ortega et al., 2009). However, Virués-Ortega et al. (2009) suggest that a two-dimension structure could be a possible factor solution. Despite the good reliability, there is a lack of evidence for tau equivalence in SCOPA-PS. Regarding divergent validity, SCOPA-PS showed a high correlation with anxiety and depression scales. No studies on ME/I across gender or culture were found.

### 3.4 Generic instruments to evaluate HRQoL in PD, PSP, and MSA

Based on the reviewed literature, the following section describes the principal generic tools used to assess HRQoL in PD, MSA, and PSP. Specifically, this section focuses on articles that included studies for these tools in PD, PSP, and MSA. The information provided here outlines the psychometric properties of the generic tools used in these patient populations.

#### 3.4.1 SF-36

The Short-Form Health Survey (SF-36) (Ware and Sherbourne, 1992) was designed to evaluate general HRQoL and includes eight scales that assess various health concepts, selected from 40 measured concepts by the Medical Outcome Study (MOS) through 36 items (Brown et al., 2009).

#### 3.4.2 SF-12

The SF-12 is the shortest form of the SF-36 questionnaire, comprising 12 items that evaluate the same eight dimensions as the SF-36. These outcomes are represented only by the PCS and MCS, with higher scores on these subscales indicating better HRQoL (Ware et al., 1996). The factor structure, reliability, convergent and divergent validity of the SF-12 in a sample of PD patients were analyzed by Jakobsson et al. (2012). The results showed good reliability for the two subscales, but the CFA outcomes showed inadequate fit indices. Conversely, Hagell and Westergren (2011) demonstrated that the structure of SF-12 showed a good fit through Item Response Theory validation, although some items showed misfits. No validation studies on PSP and MSA were found. Additionally, tau-equivalent and ME/I in PD, PSP, and MSA samples have never been investigated in any studies.

#### 3.4.3 EuroQol-5D

The EuroQol-5 (Euroqol Group, 1990) is a generic instrument used to measure the quality of life in three different ways: a descriptive system assessing health status across five dimensions, a 0–100 Visual Analogue Scale (VAS) for self-rating of one's health, and an index score reflecting the utility or preference measures. The five dimensions (5D) can be assessed using three levels (EQ-5D-3L) or five levels (EQ-5D-5L) scales or through the EQ-5D Visual Analogue Scale (VAS). The value range extends from 1.0 (“*perfect health state*”) to −1.0 (“*death*”; Martínez-Martín et al., 2003). The EQ-5D is frequently used to assess HRQoL in PD (Visser et al., 2008), PSP (Picillo et al., 2019), and MSA (Winter et al., 2011a,b), but no specific validation of this scale for these populations was found.

#### 3.4.4 NHP

The Nottingham Health Profile (NHP) is a test used to assess health status (Hunt et al., 1985). Developed in the United Kingdom, it evaluates the perception of health-related problems in physical, social, and emotional domains. The NHP is brief, easy to complete, generic, and reliable, with extensive validation of its psychometric properties across different populations (Sitzia et al., 1998; Karlsen et al., 2000). The NHP consists of two parts:

- Part 1: It contains 38 dichotomous items covering six health dimensions: *pain* (eight items), *emotional reactions* (nine items), *social isolation* (five items), *physical mobility* (eight items), *energy* (three items), and *sleep* (five items). Respondents answer “*yes*” or “*no*” to 38 questions. Each dimension is weighted, with scores ranging from 0 (“*good health*”) to 100 (“*poor health*”; Savci and Sendir, 2009). It is common for NHP-1 to be utilized alone (Savci and Sendir, 2009).
- Part 2: It consists of seven statements related to areas of daily life affected by health: *paid employment, personal relationships, jobs around the house, sex life, hobbies, interests, social life, and holidays* (Hunt et al., 1985).

For each dimension, the highest score is 100, while the generic highest score is 600; high NHP scores predict low HRQoL levels (Sitzia et al., 1998). No validation records for the NHP in Parkinson's and Parkinsonism populations were found, except one. Despite a small sample size, Hagell et al. (2003) suggested that the NHP requires further development analysis in the PD population due to some item misfits and low convergent validity with PDQ-39 subscales. Furthermore, Cronbach's alpha for the social isolation, energy, and sleep subscales was poor (0.63 < alpha < 0.78; Hagell et al., 2003), and there is no information about tau-equivalence. No ME/I studies across clinical populations and healthy subjects were found.

### 3.5 Instruments to evaluate HRQoL's predictors in PD, PSP, and MSA

In this review, we focus on the psychometric properties of HRQoL tests. Therefore, in this section, we do not discuss the psychometric properties of the tools listed below. The purpose of describing the predictor measurements is to clearly identify the construct they examine to analyze the criterion validity of HRQoL measurements. Only a qualitative description of the main impacts identified by tests and questionnaires in the literature is provided, as a meta-analytic approach would be more suitable for estimating quantitative information about the different effects.

#### 3.5.1 MDS-UPDRS

The MDS-UPDRS is the most recent version of the Unified Parkinson's Disease Rating Scale (UPDRS), originally developed in the 1980s by the Movement Disorder Society. It was designed to evaluate both MS and NMS symptoms of Parkinson's disease through a combination of clinical interviews and self-reported scales. The MDS-UPDRS items range from 0 (*normal*) to 4 (*severe*) and are organized in four subscales:

- Part 1 (nmEDL): 13 items (six semi-structured and seven self-reported) evaluate non-motor experiences of daily living.
- Part 2 (mEDL): 13 self-reported items assess motor experiences of daily living.
- Part 3 (mEx): 18 items summarize motor examination.
- Part 4 (mCompl): Six items (a semi-structured interview) evaluate motor complications.

This instrument is available in multiple languages, including English, French, Hungarian, German, Estonian, Italian, Russian, Spanish, and Slovak (Skorvanek et al., 2018; Martínez-Martín et al., 2014). In summary, the MDS-UPDRS is a multidimensional hybrid battery that assesses various characteristics related to HRQoL in PD patients.

The MDS-UPDRS was used to predict HRQoL in 26 studies (Zipprich et al., 2021; Nakano et al., 2021; Ueno et al., 2020). Our research indicates that MDS-UPDRS subscales are positively related to PDQ-39 and PDQ-8 scores, while they are negatively related to generic tests, consistent with the interpretation of the scales. In the majority of records, scholars applied linear regression to quantify the influence of disease severity on HRQoL. The results of these studies suggest a general consensus on the negative impact of both objective and self-reported motor symptoms.

However, the significance of the impact of non-motor symptoms on HRQoL varied across studies. Moreover, the proportion of HRQoL variance explained also differed across studies (e.g., Ueno et al., 2020; Simpson et al., 2014; Li et al., 2010; Grimbergen et al., 2013).

#### 3.5.2 H&Y stage scale

The Hoehn and Yahr (1998) stage is a clinical grading system based on five categories used to describe the severity of motor impairment in Parkinson's disease. The degree of motor impairment is reflected in the score, which ranges from 1 to 5. There is a consistent negative correlation between the H&Y stage and HRQoL, regardless of the statistical analysis method used. Additionally, HRQoL appears to decline progressively with increasing severity indicated by the H&Y stage, with few exceptions, such as the findings by Ophey et al. (2018) (e.g., Fereshtehnejad et al., 2014a,b; Guo et al., 2015; Hechtner et al., 2014).

#### 3.5.3 CISI-PD

The Clinical Impression of Severity Index for Parkinson's Disease is a clinical interview that assesses four dimensions related to PD symptomatology: *motor signs, disability, motor complications*, and *cognitive status* (Martinez-Martin et al., 2005).

The CISI-PD includes a motor examination, with each dimension's outcomes ranging from 0 (*no improvement*) to 6 (*very severe deficit*). Additionally, the four subscales can also be summarized into a single index, with severity levels categorized as mild (1–7), moderate (8–14), and severe (≥15). The CISI-PD scores negatively impact HRQoL, independent of the measure used.

For example, Norlin et al. (2023) demonstrated that PD patients classified according to CISI-PD sum scores displayed HRQoL levels (measured with PDQ-8 score and EQ-5D scores) corresponding to their severity. This effect appears to affect men and women across different age ranges but only moderately explains the variance in HRQoL. Disability and cognitive status showed higher effect sizes, but these results should be interpreted with caution due to the sum-score limitation of PDQ-8 and the absence of EQ-5D validation in PD populations. These effects appear consistent across the studies included, confirming the relationship between objective symptoms and self-reported HRQoL measures.

#### 3.5.4 NMS-Quest and NMSS

The Non-Motor Symptoms Questionnaire (NMS-Quest) and the Non-Motor Symptom Scale (NMSS) are tools for screening and evaluating the non-motor features of PD (Chaudhuri et al., 2007). Both tests include 30 items divided into 9 domains: c*ardiovascular, sleep disorders, mood/cognition, hallucinations, attention/memory, gastrointestinal, urinary, sexual dysfunction, and miscellaneous*.

The NMS-Quest includes 30 dichotomous items (*yes*/*no*) and is self-administrated, while the NMSS is a clinical interview in which the examiner rates the frequency and severity of symptoms reported by the patient (frequency ranges from 1 to 4 and severity from 0 to 3). The items can be summed in a general composite score or separated by domains for each questionnaire. High scores on the NMS-Quest and NMSS are associated with worse overall scores. However, replicability of the effects of non-motor symptoms on HRQoL was not consistently found across studies included in the reviews (e.g., Chaudhuri et al., 2013; Rosqvist et al., 2021; Shalash et al., 2018).

#### 3.5.5 S&E scale

The Schwab and England (1969) scale evaluates the percentage of impairment in patients' independence during activities of daily living (ranging from 100% = no impairment to 0% = completely dependent and comatose). The impact of the S&E scale on HRQoL appears independent of tools used to assess it and is strongly associated with subscales that evaluate mobility and ADL (e.g., PDQ-39 mobility and ADL subscales; Schrag et al., 2006). Moreover, the S&E scale scores correlate with psychological sunctioning and emotional wellbeing (PDQ-39 subscale and EQ-5D subscale, respectively), suggesting an association between motor impairment and these domains, further supporting a nomological network (Schrag et al., 2006).

#### 3.5.6 UMSARS

The Unified Multiple System Atrophy Rating Scales (UMSARS) is a clinical interview designed to assess the fundamental symptoms of MSA, categorizing the severity of its features on a scale from 0 (no impairment) to 4 (severe impairment; Wenning et al., 2004). The multidimensional tool includes four sections: *History of Disease* (Part I), *Motor Examination* (Part II), *Autonomic Examination* (Part III), and *Global Disability Scale* (Part IV). Only three studies have employed UMSARS as a predictor of HRQoL (Winter et al., 2011a,b; Schrag et al., 2006; Xiao et al., 2022). All these studies reached the same conclusion: there is a negative relationship between HRQoL and UMSARS scores. Specifically, UMSARS Part-II, Part-IV, and the total score appear to be strong predictors of SF-36 and EQ-5D outcomes, regardless of the type of MSA (whether cerebellar or Parkinsonian).

### 3.6 Comorbidities assessment tools

We selected the most frequent tools used for measuring psychological and psychiatric symptoms in PD, PSP, and MSA, including depression, anxiety, sleep disorder, and fatigue. The most frequent tools for depression assessment found were the Beck Depression Inventory (BDI; Beck et al., 1987), the Geriatric Depression Scale (GDS; Yesavage et al., 1982), the Hospital Anxiety and Depression Scale (HADS; Zigmond and Snaith, 1983), the Hamilton Depression Rating Scale (HAM-D; Hamilton, 1960), and the Montgomery and Asberg Depression Rating Scale (MADRS; Neumann and Schulte, 1989). Depressive symptoms consistently explained a large amount of the variance in HRQoL, regardless of the assessment tool used. For example, Santos-García and De La Fuente-Fernández (2013) found that the BDI was a good predictor of the PDQ-39 summary index, demonstrating a negative impact of depression on HRQoL (e.g., Kadastik-Eerme et al., 2015; Schrag et al., 2006; Winter et al., 2010a,b).

Regarding anxiety, several studies reported the use of the Beck Anxiety Inventory (BAI; Beck et al., 1993), HADS, and the Hamilton Anxiety Rating Scale (HAM-A; Hamilton, 1959). Similar to depression, anxiety was found to negatively impact HRQoL in PD and MSA populations (Fan et al., 2016; Meng et al., 2022; Du et al., 2018; Kovács et al., 2016). However, no information was found on anxiety's impact on HRQoL in the PSP population.

For sleep disorder evaluation, the most commonly used tools were the Epworth Sleepiness Scale (ESS; Johns, 1991) and the Parkinson's Disease Sleep Scale (PDSS; Chaudhuri et al., 2002). Although sleep disorders are addressed in NMS assessments, these two tests are specifically designed to assess sleep disorders. In the PD population, PDSS outcomes showed a negative relationship with HRQoL (Kovács et al., 2022; Liguori et al., 2021; Kovács et al., 2016; Kwon et al., 2016).

Moreover, high scores on the ESS have been shown to predict worse HRQoL outcomes in both PSP and PD populations (Shafazand et al., 2017; Kwon et al., 2013; Duncan et al., 2014; Li et al., 2023). However, no studies were found that examine the predictive value of sleep symptoms on HRQoL in MSA populations. Finally, fatigue is frequently assessed using the Fatigue Severity Scale (FSS; Krupp et al., 1989). The literature shows that higher FSS scores are associated with poorer HRQoL scores (Qin et al., 2009; Gallagher et al., 2010; Dogan et al., 2015; Sanchez-Luengos et al., 2022).

## 4 Discussion

The HRQoL is a multidimensional construct that reflects how individuals perceive their overall quality of life in relation to their health status. Measuring HRQoL is crucial for evaluating disease progression, assessing the effects of treatments, and understanding how specific symptoms impact an individual's quality of life. Therefore, it is essential that HRQoL measures are both valid and reliable. These measures should demonstrate clear psychometric properties, including a replicable latent structure across different groups (e.g., different countries, genders, etc.) and strong reliability indices (e.g., Cronbach's alpha).

In our study, we identified the most frequent tools used for assessing HRQoL across three different populations (PD, PSP, and MSA). We analyzed the psychometric properties of each tool. Furthermore, given the extensive number of studies that examined potential predictors of HRQoL, we described the most commonly used measures of these key predictors to highlight and address a potential issue of redundancy in this section.

### 4.1 Disease-specific measures

The most frequently used specific tools for assessing HRQoL in PD, PSP, and MSA are the PDQ-39, PDQ-8, PDQL, and SCOPA-PS.

#### 4.1.1 Reliability

Several records have reported generally good reliability for these instruments, but some criticisms have been raised about the procedures used to assess them. We found extensive use of Cronbach's alpha, a useful index for evaluating internal consistency, as evidence of reliability. However, the application of Cronbach's alpha requires the measurement model to be tau-equivalent and unidimensional (Flake et al., 2017). Nonetheless, no study has ever checked for tau-equivalence in any of these specific instruments. As a result, the interpretation of Cronbach's alpha may be biased due to the lack of tau-equivalence. Moreover, we found several instances where Cronbach's alpha was applied to the summary index of the PDQ-39, despite its measurement model being multi-dimensional. In conclusion, the reliability of these specific instruments remains uncertain, and the likely violation of the assumptions required for Cronbach's alpha means we cannot draw firm conclusions regarding the reliability of the PDQ-39, PDQ-8, PDQL, and SCOPA-PS.

#### 4.1.2 Construct validity 1: latent structure

We found different methods that scholars performed to determine the latent structure of these specific instruments, with principal component analysis (PCA), exploratory factor analysis (EFA), and confirmatory factor analysis (CFA) being the most frequently used. However, the literature reviewed presents inconsistent results, suggesting that the composition of subscales and the summary index of each instrument may be inappropriate. Variations in individuals' sociodemographic and cultural characteristics might explain these discrepancies. However, it is difficult to pinpoint which specific features are involved, as we did not find any records where ME/I was evaluated for these instruments across different groups.

#### 4.1.3 Construct validity 2: divergent and convergent validity

In the reviewed literature, scholars have assessed divergent and convergent validity by analyzing the correlation between tests that are theoretically designed to measure the same construct (convergent validity) or distinct constructs (divergent validity). The correlation matrices generally showed good convergent validity, with several studies reporting high correlations between specific instruments and generic ones, as well as among the specific instruments themselves. However, the PDQL social subscale showed low convergent validity, and determining the cause is difficult due to the lack of information on the latent structure and the reliability of the PDQL (de Boer et al., 1996).

However, the divergent validity of these specific instruments appears problematic, as they show high correlations with tests that assess depression, anxiety, motor symptoms (MS), and non-motor symptoms (NMS). The lack of divergent validity among these specific instruments and tests used as predictors of HRQoL is concerning, as it could lead to biased interpretations in predictive studies (Serrano-Dueñas et al., 2004; Gallagher et al., 2010).

#### 4.1.4 Recommendations

The specific instruments used to assess HRQoL in PD, PSP, and MSA could be useful tools for evaluating treatment efficacy, disease progression, and understanding how individual factors influence QoL. However, due to the limited information available on the validity and reliability of these tools, their outcomes should be interpreted with caution.

### 4.2 Generic instruments

The most frequently used generic tools for HRQoL assessment are the SF-36, SF-12, EQ-5D, and the NHP. These generic instruments are widely used across different populations. This discussion focuses on the psychometric characteristics of these instruments concerning their validity and reliability in PD, PSP, and MSA populations, aligning with the aim of the review. However, the following discussion section focuses only on the PD population, as validation studies of generic instruments specifically within PSP and MSA populations were not found.

#### 4.2.1 Reliability

The SF-36 and SF-12 generally demonstrate good reliability; however, as with the specific instruments, tau-equivalence has not been checked despite Cronbach's alpha being the principal reliability index reported.

However, the NHP displayed low Cronbach's alpha values in the social isolation, energy, and sleep subscales, indicating insufficient reliability. It is important to note that only one validation study of the NHP on a PD population was found, and the sample size was limited. Consequently, the reliability of the NHP requires further empirical evidence.

No records were found that assess the reliability of the EQ-5D in PD, MSA, and PSP populations despite its widespread use (Winter et al., 2011a,b; Schrag et al., 2006; Xiao et al., 2022). This lack of reliable data suggests that the use of EQ-5D in these populations may be biased.

#### 4.2.2 Construct validity 1: latent structure

The generic instruments discussed are self-report tools validated primarily on healthy populations, with their latent structures well-established and widely debated in the literature. However, we examined whether these latent structures remain invariant across healthy individuals and those with PD, PSP, and MSA. The invariance of latent structure is not guaranteed, and its absence could introduce significant measurement bias. We have not delved into the consequences of a lack of measurement invariance (ME/I) in various hierarchical constrained models (for more details, see Gregorich, 2006) because there are no studies addressing this issue in relation to the generic instruments. Therefore, the application of these tools to populations with PD, MSA, and PSP may be biased.

During the adaptation of tests, it could be necessary to change items significantly due to differences between the populations being studied. In such cases, a new version of the test requires a validation process as if it were a new tool. Regarding generic instruments, the few studies that evaluated the latent structure of these tests in PD, PSP, and MSA populations appear to have followed a new validation process. Only three studies have reported a CFA on SF-36, SF-12, and an application of Item Response Theory on SF-12 in the PD population (Banks and Martin, 2009; Hagell and Westergren, 2011; Jakobsson et al., 2012). The first study shows a partial replication of SF-36′s latent structure, the second suggests a mediocre fit for SF-12′s latent model, and the third indicates a misfit for some items in SF-12. These outcomes suggest that more research is needed to evaluate the psychometric properties of these instruments in PD, PSP, and MSA populations.

#### 4.2.3 Construct validity 2: divergent and convergent validity

We do not present knowledge about the divergent and convergent validity of generic instruments since it is consistent with the findings of the preceding section on the convergent and divergent validity of specific instruments.

#### 4.2.4 Recommendations

General tools for HRQoL evaluation are frequently utilized across various research paradigms, including follow-up and prediction studies based on comorbidities and symptom scales as predictors. However, we observed a major issue concerning the lack of information, particularly concerning ME/I. As a result, the findings from studies that utilize generic instruments to measure HRQoL scores should be interpreted with extreme caution.

### 4.3 Prediction models

While summarizing and analyzing predictors of HRQoL is valuable, this section focuses on whether the tools used to assess predictors are sufficiently distinct from those used to evaluate HRQoL. Through a qualitative analysis of the scales' content, we identified a possible redundancy issue that could affect some prediction models.

#### 4.3.1 MDS-UPDRS

In several studies, prediction models (e.g., through linear regression) have used the total score or specific subscales of the MDS-UPDRS as predictors of HRQoL. The self-report items of the MDS-UPDRS closely resemble those of the PDQ-39 and PDQL, raising concerns about redundancy when the total UPDRS score is used in prediction models. This overlap could explain the significance of the predictor. However, results from models that use only the UPDRS clinical interview sub-score suggest that these scales are significant predictors. It is likely that the objective symptoms evaluated through the UPDRS clinical interviews are good predictors of HRQoL, but the impact of the composite score should be interpreted with caution.

#### 4.3.2 NMS-Quest and NMSS

The Non-Motor Symptoms Scale (NMSS) is frequently identified as a significant predictor of HRQoL in various studies. However, we suspect a redundancy issue here as well, since the NMSS includes items that are very similar to those in specific and generic HRQoL rating tests. For example, Rosqvist et al. (2021) found that all NMSS subscales, including mood and attention, which contain items similar to those in the PDQ-8, were significant predictors of PDQ-8 outcomes. Similar results are reported in studies by Bugalho et al. (2021), Gan et al. (2014), and Li et al., 2010.

#### 4.3.3 H&Y

This scale is a significant predictor of HRQoL, reinforcing the idea that MSs are strong predictors of HRQoL in patients. Furthermore, the correlation between HRQoL scores and the severity of MS as rated by the H&Y scale supports the validity of HRQoL assessments, as the findings consistently show that HRQoL declines with increasing disease severity.

#### 4.3.4 CISI-PD

The subscales of the Clinical Impression of Severity Index for Parkinson's Disease (CISI-PD) are often reported as significant predictors of HRQoL. While the subscales for motor signs, cognitive status, and motor complications are conceptually distinct from HRQoL test content, the disability subscale is quite similar to specific instruments used for HRQoL assessment. The disability subscale measures impairment in activities of daily living, as rated by a clinician. Therefore, when interpreting prediction models that include the disability subscale, caution is advised, as there may be overlap with the constructs measured by HRQoL instruments.

### 4.4 Comorbidities

#### 4.4.1 Depression

Depressive symptoms are consistently associated with lower HRQoL scores, a finding that has been replicated across several studies using different tools and populations. However, these results should be interpreted with caution, as some studies do not support the divergent validity between HRQoL instruments and depression assessment tools.

Indeed, many specific and generic HRQoL tools include subscales related to “mental” wellbeing, where the items often closely resemble those found in depression assessments. This overlap raises concerns about the distinctiveness of the constructs being measured. In addition, some studies suggest that when controlling for mental HRQoL subscales, the statistical significance of depression as a predictor changes. For example, Sanchez-Luengos et al. (2022) conducted a multiple regression analysis predicting PDQ-39 outcomes using numerous variables, including the HADS. After removing the emotional wellbeing subscale, depression was no longer a significant predictor, suggesting an overlap between these features.

#### 4.4.2 Anxiety

Anxiety is negatively correlated with HRQoL, but the lack of divergent validity between anxiety tests and HRQoL instruments suggests that these results should be interpreted carefully. Furthermore, the statistical significance of anxiety as a predictor of HRQoL appears to be dependent on the assessment tools used (Kovács et al., 2016).

#### 4.4.3 Sleep disorder

Daily sleepiness and other sleep-related symptoms (e.g., insomnia) are associated with lower HRQoL scores, regardless of whether generic or specific instruments are administered. However, some studies have found no significant effect of sleep-related symptoms on HRQoL (Gallagher et al., 2010; Dogan et al., 2015). The lack of replication of sleep disorder effects may be related to issues with validity, reliability, and the lack of standardized cross-cultural adaptation processes for HRQoL tests, as discussed in the previous section.

#### 4.4.4 Fatigue

Despite some items in specific instruments, such as the mobility subscale in PDQ-39, being similar to those in the Fatigue Severity Scale (FSS), FSS consistently predicts HRQoL across different assessment tools. This suggests that increased fatigue significantly worsens HRQoL. However, this result is not supported by Tu et al. (2017a,b), which may be due to the same controversies in measurement and psychometric validation discussed in the previous sections.

### 4.5 Implication of PD interventions

Many non-pharmacological interventions for individuals with PD include psychotherapy and physical activity (Zarotti et al., 2021; Lorenzo-García et al., 2023). In the context of psychotherapy, regardless of the core theoretical approach (e.g., cognitive, behavioral, etc.), the assessment phase is crucial for two reasons: it guides the selection of the most appropriate protocol and evaluates the efficacy of the intervention after interventions.

Cognitive Behavioral Therapy (CBT) has been shown to have a positive impact on general HRQoL in PD individuals (Berardelli et al., 2015), although there are conflicting results regarding the generalizability of its effectiveness (Zarotti et al., 2021). Similarly, the literature on mindfulness interventions presents mixed findings—some studies support their effectiveness (Vandenberg et al., 2019), while others report no significant improvements in HRQoL for PD patients (Zarotti et al., 2021).

Studies on acceptance and commitment therapy (ACT) show a similar pattern, with some improvements in emotional wellbeing but no significant changes in other HRQoL subdomains (Ghielen et al., 2017; Zarotti et al., 2021). Finally, physical activities such as dancing, Tai-chi, yoga, and Qi-Gong appear to enhance general HRQoL (Lorenzo-García et al., 2023).

Our findings suggest that these discrepancies in reported effectiveness may be partly due to potential biases in the measurement tools used to evaluate non-pharmacological therapies. The principal measures used in these evaluations (e.g., PDQ-39, ESS, FSS; Zarotti et al., 2021) may be biased due to a lack of robust psychometric information or questionable reliability assessment practices. Therefore, caution is advised when interpreting the efficacy of these interventions.

## 5 Limitations

Despite the lack of psychometric information being an objective problem, the issue of redundancy emerged from a qualitative analysis of the content of HRQoL instruments and predictors' tests. While some studies support the existence of redundancy, a meta-analytic approach is required to clarify the specific effects of comorbidities and symptoms on HRQoL.

## 6 Conclusion

HRQoL is an important construct that provides valuable insights, including the fluctuations in wellbeing experienced by patients across different contexts and stages of disease progression. Consequently, the development of accurate and reliable methods for assessing HRQoL is essential for understanding how to enhance it. However, the literature analyzed in this study reveals significant gaps in the cross-cultural validation of HRQoL evaluation techniques in PD, PSP, and MSA, as well as a general lack of in-depth investigation into their psychometric properties.

One of the critical issues identified is the absence of studies exploring measurement invariance, which is essential for ensuring that HRQoL measurements are applicable across different cultural contexts and population groups. This gap raises concerns about the generalizability of research findings and the effectiveness of cultural adaptation processes for these measurement tools. The study also highlights that many HRQoL tests validated for PD populations are being applied to PSP and MSA populations, despite the lack of literature addressing ME/I across these groups. This limitation further challenges the generalizability of the findings. Another concern is the potential redundancy in the link between HRQoL measurements and the clinical aspects of PD, PSP, and MSA. Divergent validity paradigms show that the constructs designated as HRQoL subdomains are not always clearly distinguished from other psychological features, leading to poor convergence validity. This redundancy negatively impacts the criteria validity of HRQoL measures, suggesting that the variance explained by clinical characteristics might, in fact, be due to these measures evaluating overlapping constructs.

In conclusion, while HRQoL measurements are valuable tools for assessing daily living impairments and the potential influence of therapies in the clinical populations studied, there is a pressing need for further research to address the measurement components. Strengthening the psychometric properties, especially in terms of cross-cultural validity and ME/I, is essential for ensuring that these tools provide accurate and generalizable insights into the quality of life for patients with PD, PSP, and MSA.

## Data Availability

The original contributions presented in the study are included in the article/Supplementary material, further inquiries can be directed to the corresponding author.
