# Supplementary material for: Assessments scales for the evaluation of health-related quality of life in Parkinson's disease, progressive supranuclear palsy, and multiple system atrophy: a systematic review
Source: Front Psychol. 2024 Sep 10;15:1438830. doi: 10.3389/fpsyg.2024.1438830 (PMC11420144; doi:10.3389/fpsyg.2024.1438830)
Supplement: Supplementary file 1 [file Data_Sheet_1.docx]

Supplementary Materials

A’Campo, L.E.I., Wekking, E.M., Spliethoff-Kamminga, N.G.A., Le Cessie, S., and Roos, R.A.C. (2010). The benefits of a standardized patient education program for patients with Parkinson’s disease and their caregivers. *Parkinsonism and Related Disorders*, *16*(2), 89–95. <https://doi.org/10.1016/j.parkreldis.2009.07.009>

Abraham, D.S., Gruber-Baldini, A.L., Magder, L.S., McArdle, P.F., Tom, S.E., Barr, E., et al. (2019). Sex differences in Parkinson’s disease presentation and progression. *Parkinsonism and Related Disorders*, *69*, 48–54. <https://doi.org/10.1016/j.parkreldis.2019.10.019>

Ader, D.N. (2007). Developing the patient-reported outcomes measurement information system (PROMIS). Medical care, 45(5), S1-S2. <https://doi.org/10.1097/01.mlr.0000260537.45076.74>

Akbar, U., He, Y., Dai, Y., Hack, N., Malaty, I., McFarland, N.R., et al. (2015). Weight loss and impact on quality of life in Parkinson’s disease. *PLoS ONE*, *10*(5).<https://doi.org/10.1371/journal.pone.0124541>

Alvarado-Bolaños, A., Cervantes-Arriaga, A., Rodríguez-Violante, M., Llorens-Arenas, R., Calderón-Fajardo, H., Millán-Cepeda, R., et al. (2015). Convergent validation of EQ-5D-5L in patients with Parkinson’s disease. *Journal of the Neurological Sciences*, *358*(1–2), 53–57. <https://doi.org/10.1016/j.jns.2015.08.010>

Antonini, A., Bauer, L., Dohin, E., Oertel, W. H., Rascol, O., Reichmann, H., et al. (2015). Effects of rotigotine transdermal patch in patients with Parkinson’s disease presenting with non-motor symptoms - results of a double-blind, randomized, placebo-controlled trial. *European Journal of Neurology*, *22*(10), 1400–1407.<https://doi.org/10.1111/ene.12757>

Antonovsky, A., (1972). Breakdown: A needed fourth step in the conceptual armamentarium of modern medicine. *Social Science & Medicine (1967)*, *6*(5), pp.537-544. <https://doi.org/10.1016/0037-7856(72)90070-4>

Arboleda-Montealegre, G.Y., Cano-de-la-Cuerda, R., Fernández-de-las-Peñas, C., Sanchez-Camarero, C., and Ortega-Santiago, R. (2021). Drooling, swallowing difficulties and health related quality of life in parkinson’s disease patients. *International Journal of Environmental Research and Public Health*, *18*(15). <https://doi.org/10.3390/ijerph18158138>

Avidan, A., Ron Hays, M.D., Diaz, N., Bordelon, Y., Thompson, A.W., Stefanie et al. (2013). Associations of Sleep Disturbance Symptoms With Health-Related Quality of Life in Parkinson’s Disease. In *The Journal of Neuropsychiatry and Clinical Neurosciences* (Vol. 25). <https://doi.org/10.1176/appi.neuropsych.12070175>

Baig, F., Lawton, M., Rolinski, M., Ruffmann, C., Nithi, K., Evetts, S.G., et al. (2015). Delineating nonmotor symptoms in early Parkinson’s disease and first-degree relatives. *Movement Disorders*, *30*(13), 1759–1766. <https://doi.org/10.1002/mds.26281>

Balestrino, R., Hurtado-Gonzalez, C.A., Stocchi, F., Radicati, F.G., Chaudhuri, K.R., Rodriguez-Blazquez, C., et al. (2019). Applications of the European Parkinson’s Disease Association sponsored Parkinson’s Disease Composite Scale (PDCS). *Npj Parkinson’s Disease*, *5*(1). <https://doi.org/10.1038/s41531-019-0097-1>

Banks, P., and Martin, C.R. (2009). The factor structure of the SF‐36 in Parkinson's disease. Journal of Evaluation in Clinical Practice, 15(3), 460-463. <https://doi.org/10.1111/j.1365-2753.2008.01036.x>

Beck, A.T., Epstein, N., Brown, G., and Steer, R. (1993). Beck anxiety inventory. *Journal of consulting and clinical psychology*. <https://psycnet.apa.org/doi/10.1037/t02025-000>

Beck, A.T., Steer, R.A., and Brown, G.K. (1987). *Beck depression inventory*. New York:Harcourt Brace Jovanovich.

Benge, J.F., Kekecs, Z., Encarnacion, E., Ainslie, M., Herff, C., Elkins, G., et al. (2016). Duration of disease does not equally influence all aspects of quality of life in Parkinson’s disease. *Journal of Clinical Neuroscience*, *28*, 102-106. <https://doi.org/10.1016/j.jocn.2015.09.019>

Benito-León, J., Cubo, E., Coronell, C., Rodríguez-Fernández, R., Pego-Reigosa, R., Paz-González, J.M., et al. (2012). Impact of apathy on health-related quality of life in recently diagnosed Parkinson’s disease: The ANIMO study. *Movement Disorders*, *27*(2), 211–218. <https://doi.org/10.1002/mds.23872>

Berardelli, I., Pasquini, M., Bloise, M., Tarsitani, L., Biondi, M., Berardelli, A. and Fabbrini, G., (2015). CBT group intervention for depression, anxiety, and motor symptoms in Parkinson's disease: preliminary findings. *International Journal of Cognitive Therapy*, *8*(1), pp.11-20. <https://doi.org/10.1521/ijct.2015.8.1.11>

Berg, K., Wood-Dauphinee, S., and Williams, J. I. (1995). The Balance Scale: Reliability assessment with elderly residents and patients with an acute stroke. Scandinavian Journal of Rehabilitation Medicine, 27(1), 27-36.

Bergner, M., Bobbitt, R.A., Pollard, W.E., Martin, D.P., and Gilson, B.S. (1976). The Sickness Impact Profile: Validation of a Health Status Measure. Medical Care, 14(1), 57–67. <https://doi.org/10.1097/00005650-197601000-00006>

Biggs J.T., Wylie L.T. and Ziegler V.E. (1978). Validity of the Zung self-rating depression scale. Br J Psychiatry 132:381–5 <https://doi.org/10.1192/bjp.132.4.381>

Bock, M.A., Brown, E.G., Zhang, L., and Tanner, C. (2022). Association of Motor and Nonmotor Symptoms With Health-Related Quality of Life in a Large Online Cohort of People With Parkinson Disease. *Neurology*, *98*(22), E2194–E2203. <https://doi.org/10.1212/WNL.0000000000200113>

Borchani, H., Bielza, C., Martínez-Martín, P., and Larrañaga, P. (2012). Markov blanket-based approach for learning multi-dimensional Bayesian network classifiers: An application to predict the European Quality of Life-5 Dimensions (EQ-5D) from the 39-item Parkinson’s Disease Questionnaire (PDQ-39). *Journal of Biomedical Informatics*, *45*(6), 1175–1184. <https://doi.org/10.1016/j.jbi.2012.07.010>

Brazier, J., Roberts, J., and Deverill, M. (2002). The estimation of a preference-based measure of health from the SF-36. Journal of health economics, 21(2), 271-292. <https://doi.org/10.1016/S0167-6296(01)00130-8>

Brown, C.A., Cheng, E.M., Hays, R.D., Vassar, S.D., and Vickrey, B.G. (2009). SF-36 includes less Parkinson Disease (PD)-targeted content but is more responsive to change than two PD-targeted health-related quality of life measures. *Quality of Life Research*, 18(9), 1219–1237. [https://doi.org/10.1007/s11136-009-9530-y](about:blank)

Brown, K.W., and Ryan, R.M. (2003). The benefits of being present: mindfulness and its role in psychological well-being. Journal of personality and social psychology, 84(4), 822. <https://doi.org/10.1037/0022-3514.84.4.822>

Brown, R.G., Dittner, A., Findley, L., and Wessely, S.C. (2005). The Parkinson fatigue scale. Parkinsonism & related disorders, 11(1), 49-55. <https://doi.org/10.1016/j.parkreldis.2004.07.007>

Bucks, R.S., Cruise, K.E., Skinner, T.C., Loftus, A.M., Barker, R.A., and Thomas, M.G. (2011). Coping processes and health-related quality of life in Parkinson’s disease. *International Journal of GeriatricPsychiatry*, *26*(3), 247–255. <https://doi.org/10.1002/gps.2520>

Bugalho, P., Ladeira, F., Barbosa, R., Marto, J. P., Borbinha, C., da Conceição, L., et al. (2021). Progression in Parkinson’s Disease: Variation in Motor and Non-motor Symptoms Severity and Predictors of Decline in Cognition, Motor Function, Disability, and Health-Related Quality of Life as Assessed by Two Different Methods. *Movement Disorders Clinical Practice*, *8*(6), 885–895. <https://doi.org/10.1002/mdc3.13262>

Bugalho, P., Lampreia, T., Miguel, R., Mendonça, M.D., Caetano, A., and Barbosa, R. (2016). Non-Motor symptoms in Portuguese Parkinson’s Disease patients: Correlation and impact on Quality of Life and Activities of Daily Living. *Scientific Reports*, *6*. <https://doi.org/10.1038/srep32267>

Buysse, D.J., Reynolds, C.F., Monk, T.H., Berman, S.R. and Kupfer, D.J (1989). The Pittsburgh sleep quality index: a new instrument for psychiatric practice and research. Psychiatr Res 28:193–213. <https://doi.org/10.1016/0165-1781(89)90047-4>

Caap-Ahlgren, M., and Dehlin, O. (2001). Insomnia and depressive symptoms in patients with Parkinson's disease: relationship to health-related quality of life. An interview study of patients living at home. *Archives of gerontology and geriatrics*, *32*(1), 23-33 https://doi.org/10.1016/S0167-4943(00)00087-X

Candel-Parra, E., Córcoles-Jiménez, M. P., Delicado-Useros, V., Hernández-Martínez, A., and Molina-Alarcón, M. (2022). Relationship between motor and nonmotor symptoms and quality of life in patients with parkinson’s disease. *Nursing Reports*, *12*(1), 1–12. <https://doi.org/10.3390/nursrep12010001>

Candel-Parra, E., Córcoles-Jiménez, M. P., Delicado-Useros, V., Ruiz-Grao, M. C., Hernández-Martínez, A., and Molina-Alarcón, M. (2022). Predictive Model of Quality of Life in Patients with Parkinson’s Disease. *International Journal of Environmental Research and Public Health*, *19*(2). <https://doi.org/10.3390/ijerph19020672>

Cardol, M., de Haan, R. J., de Jong, B. A., van den Bos, G. A., and de Groot, I. J. (2001). Psychometric properties of the Impact on Participation and Autonomy Questionnaire. Archives of physical medicine and rehabilitation, 82(2), 210-216. <https://doi.org/10.1053/apmr.2001.18218>

Carod-Artal, F. J., Martinez-Martin, P., and Vargas, A. P. (2007). Independent validation of SCOPA-psychosocial and metric properties of the PDQ-39 Brazilian version. *Movement Disorders*, *22*(1), 91–98. <https://doi.org/10.1002/mds.21216>

Carod-Artal, F. J., Vargas, A. P., and Martinez-Martin, P. (2007). Determinants of quality of life in Brazilian patients with Parkinson’s disease. *Movement Disorders*, *22*(10), 1408–1415. <https://doi.org/10.1002/mds.21408>

Carod-Artal, F. J., Ziomkowski, S., Mourão Mesquita, H., and Martínez-Martin, P. (2008). Anxiety and depression: Main determinants of health-related quality of life in Brazilian patients with Parkinson’s disease. *Parkinsonism and Related Disorders*, *14*(2), 102–108. <https://doi.org/10.1016/j.parkreldis.2007.06.011>

Cattaneo, C., Jost, W. H., and Bonizzoni, E. (2020). Long-Term Efficacy of Safinamide on Symptoms Severity and Quality of Life in Fluctuating Parkinson’s Disease Patients. *Journal of Parkinson’s Disease*, *10*(1), 89–97. <https://doi.org/10.3233/JPD-191765>

Cella, D., Lai, J. S., Nowinski, C. J., Victorson, D., Peterman, A., Miller, D., et al. (2012). Neuro-QOL: brief measures of health-related quality of life for clinical research in neurology. *Neurology*, *78*(23), 1860–1867. <https://doi.org/10.1212/WNL.0b013e318258f744>

Chan, C. H., Chan, T. H., Leung, P. P., Brenner, M. J., Wong, V. P., Leung, E. K., et al. (2014). Rethinking well-being in terms of affliction and equanimity: Development of a holistic well-being scale. Journal of Ethnic and Cultural Diversity in Social Work, 23(3-4), 289-308. <http://dx.doi.org/10.1080/15313204.2014.932550>

Chaudhuri, KR, Martinez-Martin, P, Brown, RG, Sethi, K, Stocchi, F, Odin, P, et al. (2007). The metric properties of a novel non-motor symptoms scale for Parkinson's disease: Results from an international pilot study. Mov Disord; 22:1901–11 <https://doi.org/10.1002/mds.21596>

Chaudhuri, K.R., Rojo, J. M., Schapira, A. H., Brooks, D. J., Stocchi, F., Odin, P. et al. (2013). A proposal for a comprehensive grading of Parkinson's disease severity combining motor and non-motor assessments: meeting an unmet need. *PloS one*, *8*(2)
<https://doi.org/10.1371/journal.pone.0057221>

Chaudhuri, K. R., Pal, S., DiMarco, A., Whately-Smith, C., Bridgman, K., Mathew, R., et al. (2002). The Parkinson’s disease sleep scale: A new instrument for assessing sleep and nocturnal disability in Parkinson’s disease. Journal of Neurology Neurosurgery and Psychiatry, 73(6), 629–635. <https://doi.org/10.1136/jnnp.73.6.629>

Chaudhuri, K. R., Rizos, A., Trenkwalder, C., Rascol, O., Pal, S., Martino, D., et al. (2015). King’s Parkinson’s disease pain scale, the first scale for pain in PD: An international validation. Movement Disorders, 30(12), 1623–1631. <https://doi.org/10.1002/mds.26270>

Chekani, F., Bali, V., and Aparasu, R. R. (2016). Quality of life of patients with Parkinson’s disease and neurodegenerative dementia: A nationally representative study. *Research in Social and Administrative Pharmacy*, *12*(4), 604–613. https://doi.org/10.1016/j.sapharm.2015.09.007

Chen, K., Yang, Y. J., Liu, F. T., Li, D. K., Bu, L. L., Yang, K., et al. (2017). Evaluation of PDQ-8 and its relationship with PDQ-39 in China: A three-year longitudinal study. *Health and Quality of Life Outcomes*, *15*(1). <https://doi.org/10.1186/s12955-017-0742-5>

Cherbuin, N., and Francis Jorm, A. (2010). The informant Questionnaire on cognitive decline in the elderly (IQCODE). *Principles and practice of geriatric psychiatry*, 147-151. <https://doi.org/10.1017/s1041610204000390>

Chivers Seymour, K., Pickering, R., Rochester, L., Roberts, H. C., Ballinger, C., Hulbert, S., et al. (2019). Multicentre, randomised controlled trial of PDSAFE, a physiotherapist-delivered fall prevention programme for people with Parkinson’s. *Journal of Neurology, Neurosurgery and Psychiatry*, *90*(7), 774–782 <https://doi.org/10.1136/jnnp-2018-319448>

Chlond, M., Bergmann, F., Güthlin, C., Schnoor, H., Larisch, A., and Eggert, K. (2016). Patient education for patients with Parkinson’s disease: A randomised controlled trial. *Basal Ganglia*, *6*(1), 25–30. <https://doi.org/10.1016/j.baga.2015.11.004>

Chogahara, M. (1999). A multidimensional scale for assessing positive and negative social influences on physical activity in older adults. The Journals of Gerontology Series B: Psychological Sciences and Social Sciences, 54(6), S356-S367. <https://doi.org/10.1093/geronb/54B.6.S356>

Choi, S. M., Cho, S. H., Choe, Y., and Kim, B. C. (2023). Clinical determinants of apathy and its impact on health-related quality of life in early Parkinson disease. *Medicine (United States)*, *102*(2), E32674. <https://doi.org/10.1097/MD.0000000000032674>

Chrischilles, E.A., Rubenstein, L.M., Voelker, M.D., Wallace, R.B. and Rodnitzky, R.L., (2002). Linking clinical variables to health-related quality of life in Parkinson's disease. Parkinsonism & related disorders, 8(3), pp.199-209. <https://doi.org/10.1016/s1353-8020(01)00044-x>

Chrischilles, E. A., Rubenstein, L. M., Voelker, M. D., Wallace, R. B., and Rodnitzky, R. L. (1998). The health burdens of Parkinson’s disease. *Movement Disorders*, *13*(3), 406–413. <https://doi.org/10.1002/mds.870130306>

Chung, S. J., Calopa, M., Ceravolo, M. G., Tambasco, N., Antonini, A., Chaudhuri, K. R., et al., (2022). Effects of Levodopa-Carbidopa Intestinal Gel Compared with Optimized Medical Treatment on Nonmotor Symptoms in Advanced Parkinson’s Disease: INSIGHTS Study. *Parkinson’s Disease*, *2022*. <https://doi.org/10.1155/2022/1216975>

Chuquilín-Arista, F., Álvarez-Avellón, T., and Menéndez-González, M. (2021). Impact of depression and anxiety on dimensions of health-related quality of life in subjects with parkinson’s disease enrolled in an association of patients. *Brain Sciences*, *11*(6). <https://doi.org/10.3390/brainsci11060771>

Cubí-Mollá, P., De Vries, J., and Devlin, N. (2014). A study of the relationship between health and subjective well-being in parkinson’s disease patients. *Value in Health*, *17*(4), 372–379. <https://doi.org/10.1016/j.jval.2014.03.002>

Cummins, R. A., and Lau, A. (2006). Personal well-being index–adult. Manual, 4th edition. The.

D’Iorio, A., Vitale, C., Piscopo, F., Baiano, C., Falanga, A. P., Longo, K., et al. (2017). Impact of anxiety, apathy and reduced functional autonomy on perceived quality of life in Parkinson’s disease. *Parkinsonism and Related Disorders*, *43*, 114–117. <https://doi.org/10.1016/j.parkreldis.2017.08.003>

Damiano, A.M., McGrath, M.M., Willian, M.K., Snyder, C.F., LeWitt, P.A., Reyes, P.F., Richter, R.R. and Means, E.D., (2000). Evaluation of a measurement strategy for Parkinson's disease: assessing patient health-related quality of life. *Quality of Life Research*, *9*, pp.87-100. <https://doi.org/10.1023/a:1008928321652>

Dams, J., Klotsche, J., Bornschein, B., Reese, J. P., Balzer-Geldsetzer, M., Winter, Y., et al. (2013). Mapping the EQ-5D index by UPDRS and PDQ-8 in patients with Parkinson’s disease. *Health and Quality of Life Outcomes*, *11*(1). <https://doi.org/10.1186/1477-7525-11-35>

de Boer A. G., Wijker W., Speelman J. D., and de Haes J. C. (1996) Quality of life in patients with Parkinson’s disease: development of a questionnaire. J Neurol Neurosurg Psychiatry;61(1):70–4. <https://doi.org/10.1136/jnnp.61.1.70>

DeYoung, C. G., Quilty, L. C., and Peterson, J. B. (2007). "Between facets and domains: 10 aspects of the Big Five." *Journal of personality and social psychology* 93, no. 5: 880. <https://psycnet.apa.org/doi/10.1037/0022-3514.93.5.880>

Diaz, A. P., Freitas, F. C., de Oliveira Thais, M. E., da Silva Areas, F. Z., Schwarzbold, M. L., Debona, R., et al. (2016). Variables associated with physical health-related quality of life in Parkinson's disease patients presenting for deep brain stimulation. *Neurological sciences: official journal of the Italian Neurological Society and of the Italian Society of Clinical Neurophysiology*, *37*(11), 1831–1837. <https://doi.org/10.1007/s10072-016-2681-z>

Dodds, A. G., Bailey, P., Pearson, A., and Yates, L. (1991). Psychological factors in acquired visual impairment: The development of a scale of adjustment. Journal of Visual Impairment & Blindness, 85(7), 306-310. <https://doi.org/10.1177/0145482X9108500711>

Dogan, V. B., Koksal, A., Dirican, A., Baybas, S., Dirican, A., and Dogan, G. B. (2015). Independent effect of fatigue on health-related quality of life in patients with idiopathic Parkinson’s disease. *Neurological Sciences*, *36*(12), 2221–2226. <https://doi.org/10.1007/s10072-015-2340-9>

Du, J. J., Wang, T., Huang, P., Cui, S., Gao, C., Lin, Y., et al. (2018). Clinical characteristics and quality of life in Chinese patients with multiple system atrophy. *Brain and Behavior*, *8*(12). <https://doi.org/10.1002/brb3.1135>

Dubois, B., Burn, D., Goetz, C., Aarsland, D., Brown, R.G., Broe, G.A., et al. (2007). Diagnostic procedures for Parkinson's disease dementia: recommendations from the movement disorder society task force. *Movement disorders*, *22*(16), pp.2314-2324. <https://doi.org/10.1002/mds.21844>

Duncan, G. W., Khoo, T. K., Yarnall, A. J., O’Brien, J. T., Coleman, S. Y., Brooks, D. J., et al. (2014). Health-related quality of life in early Parkinson’s disease: The impact of nonmotor symptoms. *Movement Disorders*, *29*(2), 195–202. <https://doi.org/10.1002/mds.25664>

Duncan, P. W., Weiner, D. K., Chandler, J., and Studenski, S. (1990). Functional reach: a new clinical measure of balance. Journal of gerontology, 45(6), M192-M197. <https://doi.org/10.1093/geronj/45.6.M192>

Eglit, G. M. L., Lopez, F., Schiehser, D. M., Pirogovsky-Turk, E., Litvan, I., Lessig, S., et al. (2021). Delineation of Apathy Subgroups in Parkinson’s Disease: Differences in Clinical Presentation, Functional Ability, Health-related Quality of Life, and Caregiver Burden. *Movement Disorders Clinical Practice*, *8*(1), 92–99. <https://doi.org/10.1002/mdc3.13127>

Elbers, R. G., van Wegen, E. E., Verhoef, J., and Kwakkel, G. (2014). Impact of fatigue on health-related quality of life in patients with Parkinson’s disease: a prospective study. *Clinical Rehabilitation*, *28*(3), 300-311. <https://doi.org/10.1177/0269215513503355>

Ellis, T., Cavanaugh, J. T., Earhart, G. M., Ford, M. P., Foreman, K. B., and Dibble, L. E. (2011). Which measures of physical function and motor impairment best predict quality of life in Parkinson’s disease? *Parkinsonism and Related Disorders*, *17*(9), 693– 697. <https://doi.org/10.1016/j.parkreldis.2011.07.004>

Endler, N., and Parker, J. D. (1999). Coping inventory for stressful situations.<https://doi.org/10.1037/t13031-000>

Erga, A. H., Alves, G., Tysnes, O. B., and Pedersen, K. F. (2020). Impulsive and compulsive behaviors in Parkinson’s disease: Impact on quality of and satisfaction with life, and caregiver burden. *Parkinsonism and Related Disorders*, *78*, 27–30. <https://doi.org/10.1016/j.parkreldis.2020.07.007>

Fahn, S.R.L.E., (1987). Unified Parkinson's disease rating scale. *Recent developments in Parkinson's disease*, pp.153-163.

Fan, J. Y., Chang, B. L., and Wu, Y. R. (2016). Relationships among Depression, Anxiety, Sleep, and Quality of Life in Patients with Parkinson’s Disease in Taiwan. *Parkinson’s Disease*, *2016*. <https://doi.org/10.1155/2016/4040185>

Fan, X., Wang, D., Hellman, B., Janssen, M. F., Bakker, G., Coghlan, R., et al. (2018). Assessment of health-related quality of life between people with parkinson’s disease and non-parkinson’s: Using data drawn from the ‘100 for parkinson’s’ smartphone-based prospective study. *International Journal of Environmental Research and Public Health*, *15*(11). <https://doi.org/10.3390/ijerph15112538>

Farhadi, F., Vosoughi, K., Shahidi, G. A., Delbari, A., Lökk, J., and Fereshtehnejad, S. M. (2017). Sexual dimorphism in Parkinson’s disease: Differences in clinical manifestations, quality of life and psychosocial functioning between males and females. *Neuropsychiatric Disease and Treatment*, *13*, 329–338. <https://doi.org/10.2147/NDT.S124984>

Fereshtehnejad, S. M., Farhadi, F., Hadizadeh, H., Shahidi, G. A., Delbari, A., and Lökk, J. (2014). Cross-cultural validity, reliability, and psychometric properties of the persian version of the scales for outcomes in parkinson’s disease-psychosocial questionnaire. *Neurology Research International*, *2014*. <https://doi.org/10.1155/2014/260684>

Fereshtehnejad, S. M., Naderi, N., Rahmani, A., Shahidi, G. A., Delbari, A., and Lökk, J. (2014). Psychometric study of the Persian short-form eight-item Parkinson’s disease questionnaire (PDQ-8) to evaluate health related quality of life (HRQoL). *Health and Quality of Life Outcomes*, *12*(1). <https://doi.org/10.1186/1477-7525-12-78>

Fereshtehnejad, S. M., Shafieesabet, M., Farhadi, F., Hadizadeh, H., Rahmani, A., Naderi, N., et al. (2015). Heterogeneous determinants of quality of life in different phenotypes of Parkinson’s disease. *PLoS ONE*, *10*(9). <https://doi.org/10.1371/journal.pone.0137081>

Ferguson, L., and Scheman, J. (2009). Patient global impression of change scores within the context of a chronic pain rehabilitation program. *The Journal of Pain*, *10*(4), S73. <https://doi.org/10.1016/j.jpain.2009.01.258>

Fillenbaum, G. G., and Smyer, M. A. (1981). The development, validity, and reliability of the OARS multidimensional functional assessment questionnaire. Journal of gerontology, 36(4), 428-434. <https://doi.org/10.1093/geronj/36.4.428>

Fitzpatrick, R., Norquist, J. M., and Jenkinson, C. (2004). Distribution-based criteria for change in health-related quality of life in Parkinson’s disease. *Journal of Clinical Epidemiology*, *57*(1), 40–44. <https://doi.org/10.1016/j.jclinepi.2003.07.003>

Fitzpatrick, R., Peto, V., Jenkinson, C., Greenhall, R., and Hyman, N. (1997). Health-related quality of life in Parkinson’s disease: A study of outpatient clinic attenders. *Movement Disorders*, *12*(6), 916–922. <https://doi.org/10.1002/mds.870120613>

Flake, J. K., Pek, J., and Hehman, E. (2017). Construct validation in social and personality research: Current practice and recommendations. *Social Psychological and Personality Science*, *8*(4), 370-378. <https://doi.org/10.1177/1948550617693063>

Folkman, S., and Lazarus, R. S. (1985). If it changes it must be a process: study of emotion and coping during three stages of a college examination. Journal of personality and social psychology, 48(1), 150. <https://doi.org/10.1037/0022-3514.48.1.150>

Forsaa, E. B., Larsen, J. P., Wentzel-Larsen, T., Herlofson, K., and Alves, G. (2008). Predictors and course of health-related quality of life in Parkinson’s disease. *Movement Disorders*, *23*(10), 1420–1427. <https://doi.org/10.1002/mds.22121>

Franchignoni, F., Giordano, A., and Ferriero, G. (2008). Rasch analysis of the short form 8-item Parkinson’s Disease Questionnaire (PDQ-8). *Quality of Life Research*, *17*(4), 541–548. <https://doi.org/10.1007/s11136-008-9341-6>

Franke, G.H., Mähner, N., Reimer, J., Spangemacher, B. and Esser, J., (2000). Erste Überprüfung des Essener Fragebogens zur Krankheitsverarbeitung (EFK) an sehbeeinträchtigten Patienten. [https://doi.org/10.1024//0170-1789.21.2.166](https://doi.org/10.1024/0170-1789.21.2.166)

Franke, G.H.; Nentzl, J., and Jagla-Franke, M. (2020) SAMS. Stendal Adherence to Medication Score. Available online: https://www.psychometrikon.de/inhalt/suchen/test.php?id=ff32ee9ea015021c3fb047e505e2bc45 (accessed on 22 February 2021).

Furlong, W., Feeny, D., Torrance, G., Goldsmith, C., DePauw, S., Zhu, Z., et al. (1998). *Multiplicative multi-attribute utility function for the Health Utilities Index Mark 3 (HUI3) system: a technical report* (No. 1998-11). Centre for Health Economics and Policy Analysis (CHEPA), McMaster University, Hamilton, Canada.

Galeoto, G., Colalelli, F., Massai, P., Berardi, A., Tofani, M., Pierantozzi, M., et al. (2018). Quality of life in Parkinson’s disease: Italian validation of the Parkinson’s Disease Questionnaire (PDQ-39-IT). *Neurological Sciences*, *39*(11), 1903–1909. <https://doi.org/10.1007/s10072-018-3524-x>

Gallagher, D. A., Lees, A. J., and Schrag, A. (2010). What are the most important nonmotor symptoms in patients with Parkinson’s disease and are we missing them? *Movement Disorders*, *25*(15), 2493–2500. <https://doi.org/10.1002/mds.23394>

Gan, J., Zhou, M., Chen, W., and Liu, Z. (2014). Non-motor symptoms in Chinese Parkinson’s disease patients. *Journal of Clinical Neuroscience*, *21*(5), 751–754. <https://doi.org/10.1016/j.jocn.2013.07.015>

García-Gordillo, M. Á., del Pozo-Cruz, B., Adsuar, J. C., Cordero-Ferrera, J. M., Abellán-Perpiñán, J. M., and Sánchez-Martínez, F. I. (2015). Validación y comparación de los instrumentos EQ-5D-3L y SF-6D en una muestra de población española con enfermedad de Parkinson. *Nutrición Hospitalaria*, *32*(6), 2808–2821. <https://doi.org/10.3305/nh.2015.32.6.9765>

García-Gordillo, M. Á., del Pozo-Cruz, B., Adsuar, J. C., Sánchez-Martínez, F. I., and Abellán-Perpiñán, J. M. (2014). Validation and comparison of 15-D and EQ-5D-5L instruments in a Spanish Parkinson’s disease population sample. *Quality of Life Research: An International Journal of Quality of Life Aspects of Treatment, Care and Rehabilitation*, *23*(4), 1315–1326. <https://doi.org/10.1007/s11136-013-0569-4>

Gazibara, T., Kisic-Tepavcevic, D., Svetel, M., Tomic, A., Stankovic, I., Kostic, V. S., et al. (2016). Health-related quality of life as a predictor of recurrent falling in Parkinson’s disease: 1-year follow-up study. *Psychogeriatrics*, *16*(6), 362–367. <https://doi.org/10.1111/psyg.12178>

Gazibara, T., Pekmezovic, T., KisicTepavcevic, D., Svetel, M., Tomic, A., Stankovic, I., et al. (2015). Health-related quality of life in patients with Parkinson’s disease: Implications for falling. *Parkinsonism and Related Disorders*, *21*(6), 573–576. <https://doi.org/10.1016/j.parkreldis.2015.03.007>

Ghielen, I., van Wegen, E.E., Rutten, S., de Goede, C.J., Houniet-de Gier, M., Collette, E.H. et al. (2017). Body awareness training in the treatment of wearing-off related anxiety in patients with Parkinson's disease: Results from a pilot randomized controlled trial. *Journal of Psychosomatic Research*, *103*, .1-8. <https://doi.org/10.1016/j.jpsychores.2017.09.008>

Ghorbani Saeedian, R., Nagyova, I., Klein, D., Skorvanek, M., Rosenberger, J., Gdovinova, Z., et al. (2014). Self‐rated health mediates the association between functional status and health‐related quality of life in Parkinson's disease. *Journal of clinical nursing*, *23*(13-14), 1970-1977. <https://doi.org/10.1111/jocn.12442>

Giladi, N., Shabtai, H., Simon, E. S., Biran, S., Tal, J., and Korczyn, A. D. (2000). Construction of freezing of gait questionnaire for patients with Parkinsonism. *Parkinsonism & related disorders*, *6*(3), 165–170. <https://doi.org/10.1016/s1353-8020(99)00062-0>

Gison, A., Rizza, F., Bonassi, S., Dall’Armi, V., Lisi, S., and Giaquinto, S. (2014). The sense-of-coherence predicts health-related quality of life and emotional distress but not disability in Parkinson’s disease. *BMC Neurology*, *14*(1). <https://doi.org/10.1186/s12883-014-0193-0>

Global Parkinson's Disease Survey (GPDS) Steering Committee.. Factors impacting on quality of life in Parkinson's disease: results from an international survey. Mov Disord. (2002) Jan;17(1):60-7. <https://doi.org/10.1002/mds.10010>

Goetz, C. G., Stebbins, G. T., Shale, H. M., Lang, A. E., Chernik, D. A., Chmura, T. A., et al. (1994). Utility of an objective dyskinesia rating scale for Parkinson's disease: inter- and intrarater reliability assessment. *Movement disorders: official journal of the Movement Disorder Society*, *9*(4), 390–394. <https://doi.org/10.1002/mds.870090403>

Goetz, C. G., Tilley, B. C., Shaftman, S. R., Stebbins, G. T., Fahn, S., Martinez-Martin, P., et al. (2008). Movement Disorder Society-sponsored revision of the Unified Parkinson's Disease Rating Scale (MDS-UPDRS): scale presentation and clinimetric testing results. *Movement disorders: official journal of the Movement Disorder Society*, *23*(15), 2129–2170. <https://doi.org/10.1002/mds.22340>

Golbe, L. I., and Ohman-Strickland, P. A. (2007). A clinical rating scale for progressive supranuclear palsy. *Brain : a journal of neurology*, *130*(Pt 6), 1552–1565. <https://doi.org/10.1093/brain/awm032>

Grandas, F., and Hernández, B. (2007). Long-term effectiveness and quality of life improvement in entacapone-treated Parkinson’s disease patients: The effects of an early therapeutic intervention. *European Journal of Neurology*, *14*(3), 282–289. <https://doi.org/10.1111/j.1468-1331.2006.01635.x>

Gregorich S. E. (2006). Do self-report instruments allow meaningful comparisons across diverse population groups? Testing measurement invariance using the confirmatory factor analysis framework. *Medical care*, *44*(11 Suppl 3), S78–S94. <https://doi.org/10.1097/01.mlr.0000245454.12228.8f>

Grimbergen, Y. A., Schrag, A., Mazibrada, G., Borm, G. F., and Bloem, B. R. (2013). Impact of falls and fear of falling on health-related quality of life in patients with Parkinson's disease. *Journal of Parkinson's disease*, *3*(3), 409-413. https://doi.org/10.3233/JPD-120113

Grosset, D., Taurah, L., Burn, D. J., MacMahon, D., Forbes, A., Turner, K., et al. (2007). A multicentre longitudinal observational study of changes in self reported health status in people with Parkinson's disease left untreated at diagnosis. *Journal of neurology, neurosurgery, and psychiatry*, *78*(5), 465–469. <https://doi.org/10.1136/jnnp.2006.098327>

Group, T. E. (1990). EuroQol-a new facility for the measurement of health-related quality of life. Health policy, 16(3), 199-208.<https://doi.org/10.1016/0168-8510(90)90421-9>

Gruber, M. T., Witte, O. W., Grosskreutz, J., and Prell, T. (2020). Association between malnutrition, clinical parameters and health-related quality of life in elderly hospitalized patients with Parkinson’s disease: A cross-sectional study. *PLoS ONE*, *15*(5). <https://doi.org/10.1371/journal.pone.0232764>

Guo, X., Song, W., Chen, K., Chen, X., Zheng, Z., Cao, B., et al. (2015). Impact of Frontal Lobe Function and Behavioral Changes on Health-Related Quality of Life in Patients with Parkinson’s Disease: A Cross-Sectional Study from Southwest China. *European Neurology*, *74*(3–4), 147–153. <https://doi.org/10.1159/000439084>

Haapaniemi, T. H., Sotaniemi, K. A., Sintonen, H., and Taimela, E. (2004). The generic 15D instrument is valid and feasible for measuring health related quality of life in Parkinson’s disease. *Journal of Neurology, Neurosurgery and Psychiatry*, *75*(7), 976– 983. <https://doi.org/10.1136/jnnp.2003.015693>

Hagell, P., and Westergren, A. (2011). Measurement properties of the SF-12 health survey in Parkinson's disease. *Journal of Parkinson's disease*, *1*(2), 185–196. <https://doi.org/10.3233/JPD-2011-11026>

Hagell, P., Whalley, D., McKenna, S. P., and Lindvall, O. (2003). Health status measurement in Parkinson's disease: validity of the PDQ‐39 and Nottingham Health Profile. Movement Disorders, 18(7), 773-783. <https://doi.org/10.1002/mds.10438>

Hamilton M. (1959) The assessment of anxiety states by rating. Br J Med Psychol 32:50–55 <https://doi.org/10.1111/j.2044-8341.1959.tb00467.x>

Hamilton, M. (1960). A rating scale for depression. Journal of neurology, neurosurgery, and psychiatry, 23(1), 56. doi: 10.1136/jnnp.23.1.56

Hanna, K. K., and Cronin-Golomb, A. (2012). Impact of anxiety on quality of life in parkinson’s disease. *Parkinson’s Disease*. <https://doi.org/10.1155/2012/640707>

Hattori, N., Takeda, A., Hanya, Y., Kitagawa, T., Arai, M., Furusawa, Y., et al. (2022). Effects of rasagiline on Parkinson’s Disease Questionnaire (PDQ-39) emotional well-being domain in patients with Parkinson’s disease: A post-hoc analysis of clinical trials in Japan. *PLoS ONE*, *17*(1 1).<https://doi.org/10.1371/journal.pone.0262796>

He, L., Lee, E. Y., Sterling, N. W., Kong, L., Lewis, M. M., Du, G., et al. (2016). The key determinants to quality of life in Parkinson’s disease patients: Results from the Parkinson’s disease biomarker program (PDBP). *Journal of Parkinson’s Disease*, *6*(3), 523–532. <https://doi.org/10.3233/JPD-160851>

Hechtner, M. C., Vogt, T., Zöllner, Y., Schröder, S., Sauer, J. B., Binder, H., et al. (2014). Quality of life in Parkinson’s disease patients with motor fluctuations and dyskinesias in five European countries. *Parkinsonism and Related Disorders*, *20*(9), 969–974. <https://doi.org/10.1016/j.parkreldis.2014.06.001>

Herlofson, K., and Larsen, J. P. (2003). The influence of fatigue on health-related quality of life in patients with Parkinson’s disease. *Acta Neurologica Scandinavica*, *107*(1), 1–6. <https://doi.org/10.1034/j.1600-0404.2003.02033.x>

Herman, T., Weiss, A., Brozgol, M., Wilf-Yarkoni, A., Giladi, N., and Hausdorff, J. M. (2015). Cognitive function and other non-motor features in non-demented Parkinson’s disease motor subtypes. *Journal of Neural Transmission*, *122*(8), 1115–1124. <https://doi.org/10.1007/s00702-014-1349-1>

Higgins, J.P. and Green, S. (2011) Cochrane Handbook for Systematic Reviews of Interventions. 4th ed. Chichester: John Wiley & Sons.

Hoehn, M. M., and Yahr, M. D. (1967). Parkinsonism: onset, progression and mortality. *Neurology*, *17*(5), 427–442. <https://doi.org/10.1212/wnl.17.5.427>

Hoehn M. M., and Yahr M.D (1998). [Parkinsonism: onset, progression, and mortality](https://citeseerx.ist.psu.edu/viewdoc/download?doi=10.1.1.455.6557&rep=rep1&type=pdf). Neurology. 50(2):318.

Horak, F. B., Wrisley, D. M., and Frank, J. (2009). The balance evaluation systems test (BESTest) to differentiate balance deficits. Physical therapy, 89(5), 484-498. <https://doi.org/10.2522/ptj.20080071>

Hristova, D. R., Hristov, J. I., Mateva, N. G., and Papathanasiou, J. V. (2009). Quality of life in patients with Parkinson's disease. *Folia medica*, *51*(4), 58.

Huang, T. T., Hsu, H. Y., Wang, B. H., and Chen, K. H. (2011). Quality of life in Parkinson’s disease patients: validation of the Short-Form Eight-item Parkinson’s Disease Questionnaire (PDQ-8) in Taiwan. *Quality of Life Research: An International Journal of Quality of Life Aspects of Treatment, Care and Rehabilitation*, *20*(4), 499–505. <https://doi.org/10.1007/s11136-010-9777-3>

Hunt, S.M., McEwen, J. and McKenna, S.P., (1985). Measuring health status: a new tool for clinicians and epidemiologists. *The Journal of the Royal College of General Practitioners*, *35*(273), pp.185-188.

Hurst, H., and Bolton, J. (2004). Assessing the clinical significance of change scores recorded on subjective outcome measures. *Journal of manipulative and physiological therapeutics*, *27*(1), 26–35. <https://doi.org/10.1016/j.jmpt.2003.11.003>

Hurt, C. S., Burn, D. J., Hindle, J., Samuel, M., Wilson, K., and Brown, R. G. (2014). Thinking positively about chronic illness: An exploration of optimism, illness perceptions and well-being in patients with Parkinson’s disease. *British Journal of Health Psychology*, 19(2), 363–379. <https://doi.org/10.1111/bjhp.12043>

Hurt, C. S., Landau, S., Burn, D. J., Hindle, J. V., Samuel, M., Wilson, K., et al. (2012). Cognition, coping, and outcome in Parkinson’s disease. *International Psychogeriatrics*, *24*(10), 1656–1663. <https://doi.org/10.1017/S1041610212000749>

Iansek, R., and Danoudis, M. (2020). Patients’ Perspective of Comprehensive Parkinson Care in Rural Victoria. *Parkinson’s Disease*, *2020*. <https://doi.org/10.1155/2020/2679501>

Jahanshahi M., and Marsden, C.D. (1988) Personality in torticollis: a controlled study. Psychol Med ;18:375–387 <https://doi.org/10.1017/s0033291700007923>

Jakobsson, U., Westergren, A., Lindskov, S., and Hagell, P. (2012). Construct validity of the SF-12 in three different samples. *Journal of evaluation in clinical practice*, *18*(3), 560–566. <https://doi.org/10.1111/j.1365-2753.2010.01623.x>

Järvelä, J. T., and Kaasinen, V. (2016). Pharmacotherapy and generic health-related quality of life in Parkinson’s disease. *Acta Neurologica Scandinavica*, *134*(3), 205–209. <https://doi.org/10.1111/ane.12531>

Jecmenica-Lukic, M.V., Pekmezovic, T.D., Petrovic, I.N., Dragasevic, N.T. and Kostić, V.S., (2018). Factors associated with deterioration of health-related quality of life in multiple system atrophy: 1-year follow-up study. *Acta Neurologica Belgica*, *118*, pp.589-595.

Jenkinson, C., Fitzpatrick, R., Peto, V., Greenhall, R., and Hyman, N. (1997). The PDQ-8: development and validation of a short-form Parkinson's disease questionnaire. *Psychology and Health*, *12*(6), 805-814. http://doi.org/https:/doi.org/10.1007/bf02260863

Jette, A. M., Davies, A. R., Cleary, P. D., Calkins, D. R., Rubenstein, L. V., Fink, A., et al. (1986). The Functional Status Questionnaire: reliability and validity when used in primary care. *Journal of general internal medicine*, *1*(3), 143–149. <https://doi.org/10.1007/BF02602324>

Jeyadevan, A., Bakeberg, M., Byrnes, M., Kenna, J., McGregor, S., Ghosh, S., et al. (2023). Quality of life implications for elevated trait impulsivity in people with Parkinson’s disease. *Quality of Life Research*, *32*(4), 1143–1150. <https://doi.org/10.1007/s11136-022-03321-w>

Johns M. W. (1991). A new method for measuring daytime sleepiness: the Epworth sleepiness scale. *Sleep*, *14*(6), 540–545. <https://doi.org/10.1093/sleep/14.6.540>

Jones, C. A., Pohar, S. L., and Patten, S. B. (2009). Major depression and health-related quality of life in Parkinson’s disease. *General Hospital Psychiatry*, *31*(4), 334–340. <https://doi.org/10.1016/j.genhosppsych.2009.03.009>

Jones, J. D., Hass, C., Mangal, P., Lafo, J., Okun, M. S., and Bowers, D. (2014). The Cognition and Emotional Well-being indices of the Parkinson’s disease questionnaire-39: What do they really measure? *Parkinsonism and Related Disorders*, *20*(11), 1236–1241. <https://doi.org/10.1016/j.parkreldis.2014.09.014>

Joseph, C., Jonsson-Lecapre, J., Wicksell, R., Svenningsson, P., and Franzén, E. (2019). Pain in persons with mild-moderate Parkinson’s disease: a cross-sectional study of pain severity and associated factors. *International Journal of Rehabilitation Research. Internationale Zeitschrift Fur Rehabilitationsforschung. Revue Internationale de Recherches de Readaptation*, *42*(4), 371–376. <https://doi.org/10.1097/MRR.0000000000000373>

Josiah, A. F., Gruber-Baldini, A. L., Anderson, K. E., Fishman, P. S., Weiner, W. J., Reich, S. G., et al. (2012). The effects of gait impairment with and without freezing of gait in Parkinson’s disease. *Parkinsonism and Related Disorders*, *18*(3), 239–242. <https://doi.org/10.1016/j.parkreldis.2011.10.008>

Just, H. and Ostergaard, K. (2002). Health-related quality of life in patients with advanced Parkinson’s disease treated with deep brain stimulation of the subthalamic nuclei. *Movement Disorders*, *17*(3), 539–545. <https://doi.org/10.1002/mds.10111>

Kadastik-Eerme, L., Rosenthal, M., Paju, T., Muldmaa, M., and Taba, P. (2015). Health-related quality of life in Parkinson’s disease: A cross-sectional study focusing on non-motor symptoms. *Health and Quality of Life Outcomes*, *13*(1). https://doi.org/10.1186/s12955-015-0281-x

Kahraman, T., Genç, A., Söke, F., Göz, E., DönmezÇolakoğlu, B., and Keskinoğlu, P. (2018). Validity and reliability of the turkish version of the 8-item parkinson’s disease questionnaire. *Noropsikiyatri Arsivi*, *55*(4), 337–340 <https://doi.org/10.5152/npa.2017.19343>

Karlsen, K. H., Larsen, J. P., Tandberg, E., and Maeland, J. G. (1999). Influence of clinical and demographic variables on quality of life in patients with Parkinson’s disease. In *J Neurol Neurosurg Psychiatry* (Vol. 66). <https://doi.org/10.1136/jnnp.66.4.431>

Karlsen, K. H., Larsen, J. P., Tandberg, E., and Mæland, J. G. (1998). Quality of life measurements in patients with Parkinson’s disease: A community-based study. *European Journal of Neurology*, *5*(5), 443–450. <https://doi.org/10.1046/j.1468-1331.1998.550443.x>

Karlsen, K. H., Tandberg, E., Årsland, D., and Larsen, J. P. (2000). Health related quality of life in Parkinson’s disease: A prospective longitudinal study. *Journal of Neurology Neurosurgery and Psychiatry*, *69*(5), 584–589. <https://doi.org/10.1136/jnnp.69.5.584>

Karlstedt, M., Fereshtehnejad, S. M., Aarsland, D., and Lökk, J. (2018). Mediating effect of mutuality on health-related quality of life in patients with Parkinson’s disease. *Parkinson’s Disease*, *2018*. <https://doi.org/10.1155/2018/9548681>

Katsarou, Z., Bostantjopoulou, S., Peto, V., Alevriadou, A., and Kiosseoglou, G. (2001). Quality of life in Parkinson's disease: Greek translation and validation of the Parkinson's disease questionnaire (PDQ-39). *Quality of Life Research*, *10*, 159-163 https://doi.org/10.1023/A:1016720400862

Katsarou, Z., Bostantjopoulou, S., Peto, V., Kafantari, A., Apostolidou, E., and Peitsidou, E. (2004). Assessing quality of life in Parkinson's disease: can a short‐form questionnaire be useful?.*Movement disorders: official journal of the Movement Disorder Society*, *19*(3), 308-312. <https://doi.org/10.1002/mds.10678>

Kellerborg, K., Norlin, J. M., and Odin, P. (2023). The Relationship between PDQ-8 and Costs in Parkinson’s Disease—A Swedish Register-Based Study. *Movement Disorders Clinical Practice*, *10*(2), 231–237. <https://doi.org/10.1002/mdc3.13630>

Kenna, J. E., Bakeberg, M. C., Abonnel, M. Y., Mastaglia, F. L., and Anderton, R. S. (2022). Impact of Gastrointestinal Symptoms on Health-Related Quality of Life in an Australian Parkinson’s Disease Cohort. *Parkinson’s Disease*, *2022* https://doi.org/10.1155/2022/4053665

Kertesz, A., Davidson, W., and Fox, H. (1997). Frontal behavioral inventory: diagnostic criteria for frontal lobe dementia. Canadian Journal of Neurological Sciences, 24(1), 29-36. <https://doi.org/10.1017/s0317167100021053>

Kessler, R. C., Andrews, G., Mroczek, D., Ustun, B., and Wittchen, H. U. (1998). The World Health Organization composite international diagnostic interview short‐form (CIDI‐SF). *International journal of methods in psychiatric research*, *7*(4), 171-185. <https://doi.org/10.1002/mpr.47>

Kim, Y. E., Kim, H. J., Yun, J. Y., Lee, W. W., Yang, H. J., Kim, J. M., et al. (2018). *Musculoskeletal Problems Affect the Quality of Life of Patients with Parkinson’s Disease*. <https://doi.org/10.14802/jmd.18022/J>

Klepac, N., and Trkulja, V. (2009). Education effect on depression and quality of life in nondemented Parkinson's disease patients. *The Journal of neuropsychiatry and clinical neurosciences*, *21*(3), 314–322. <https://doi.org/10.1176/jnp.2009.21.3.314>

Klepac, N., Hajnšek, S., and Trkulja, V. (2010). Impact of pre-morbid depression on health-related quality of life in non-demented Parkinson’s disease patients. *Parkinsonism and Related Disorders*, *16*(1), 21–27. <https://doi.org/10.1016/j.parkreldis.2009.07.003>

Klepac, N., Trkulja, V., Relja, M., and Babić, T. (2008). Is quality of life in non-demented Parkinson’s disease patients related to cognitive performance? A clinic-based cross-sectional study. *European Journal of Neurology*, *15*(2), 128–133. <https://doi.org/10.1111/j.1468-1331.2007.02011.x>

Klietz, M., Schnur, T., Drexel, S., Lange, F., Tulke, A., Rippena, L., et al. (2020). Association of Motor and Cognitive Symptoms with Health-Related Quality of Life and Caregiver Burden in a German Cohort of Advanced Parkinson’s Disease Patients. *Parkinson’s Disease*, *2020*. <https://doi.org/10.1155/2020/5184084>

Klotsche, J., Reese, J. P., Winter, Y., Oertel, W. H., Irving, H., Wittchen, H. U., et al. (2011). Trajectory classes of decline in health-related quality of life in parkinson’s disease: A pilot study. *Value in Health*, *14*(2), 329–338. <https://doi.org/10.1016/j.jval.2010.10.005>

Koutsouras, G. W., Levine, K., Duroseau, N., Ciraco, C., Chan, V., Pergament, K., et al. (2020). Effects of depression and exercise on health-related quality of life in patients with Parkinson’s disease. *Chronic Illness*, *16*(3), 190–200. <https://doi.org/10.1177/1742395318796166>

Kovács, M., Makkos, A., Aschermann, Z., Janszky, J., Komoly, S., Weintraut, R., et al. (2016). Impact of Sex on the Nonmotor Symptoms and the Health-Related Quality of Life in Parkinson’s Disease. *Parkinson’s Disease*, *2016*. <https://doi.org/10.1155/2016/7951840>

Kovács, N., Bergmann, L., Anca-Herschkovitsch, M., Cubo, E., Davis, T. L., Iansek, R., et al. (2022). Outcomes Impacting Quality of Life in Advanced Parkinson’s Disease Patients Treated with Levodopa-Carbidopa Intestinal Gel. *Journal of Parkinson’sDisease*, *12*(3), 917–926. <https://doi.org/10.3233/JPD-212979>

Kroenke, K., Spitzer, R. L., and Williams, J. B. (2001). The PHQ‐9: validity of a brief depression severity measure. Journal of general internal medicine, 16(9), 606-613.
<https://doi.org/10.1046/j.1525-1497.2001.016009606.x>

Kroll T, Kehn M, Ho PS, and Groah S. (2007) The SCI Exercise Self-Efficacy Scale (ESES): development and psychometric properties. Int J BehavNutr Phys Act. 2007 Aug 30;4:34. doi:10.1186/1479-5868-4-34

Krupp, L. B., LaRocca, N. G., Muir-Nash, J., and Steinberg, A. D. (1989). The fatigue severity scale: application to patients with multiple sclerosis and systemic lupus erythematosus. Archives of neurology, 46(10), 1121-1123. doi:10.1001/archneur.1989.00520460115022

Kuhlman, G. D., Flanigan, J. L., Sperling, S. A., and Barrett, M. J. (2019). Predictors of health-related quality of life in Parkinson’s disease. *Parkinsonism and Related Disorders*, *65*, 86–90. <https://doi.org/10.1016/j.parkreldis.2019.05.009>

Kurt, E. E., Büyükturan, B., Büyükturan, Ö., Erdem, H. R., and Tuncay, F. (2018). Effects of Ai Chi on balance, quality of life, functional mobility, and motor impairment in patients with Parkinson’s disease*. *Disability and Rehabilitation*, *40*(7), 791–797. <https://doi.org/10.1080/09638288.2016.1276972>

Kwok, J. Y. Y., Auyeung, M., and Chan, H. Y. L. (2020). Examining factors related to health-related quality of life in people with Parkinson’s disease. *Rehabilitation Nursing Journal*, *45*(3), 122-130. <https://doi.org/10.1097/rnj.0000000000000179>

Kwok, J. Y. Y., Choi, E. P. H., Chau, P. H., Wong, J. Y. H., Fong, D. Y. T., and Auyeung, M. (2020). Effects of spiritual resilience on psychological distress and health-related quality of life in Chinese people with Parkinson’s disease. *Quality of Life Research*, *29*(11), 3065–3073. <https://doi.org/10.1007/s11136-020-02562-x>

Kwok, J. Y. Y., Choi, E. P. H., Lee, J. J., Lok, K. Y. W., Kwan, J. C. Y., Mok, V. C. T., et al. (2022). Effects of Mindfulness Yoga Versus Conventional Physical Exercises on Symptom Experiences and Health-related Quality of Life in People with Parkinson’s Disease: The Potential Mediating Roles of Anxiety and Depression. *Annals of Behavioral Medicine*, *56*(10), 1068–1081. <https://doi.org/10.1093/abm/kaac005>

Kwon, D. Y., Kim, J. W., Ma, H. Il, Ahn, T. B., Cho, J., Lee, P. H., et al. (2013). Translation and validation of the Korean version of the 39-item Parkinson’s disease questionnaire. *Journal of Clinical Neurology (Korea)*, *9*(1), 26–31.<https://doi.org/10.3988/jcn.2013.9.1.26>

Kwon, D. Y., Koh, S. B., Lee, J. H., Park, H. K., Kim, H. J., Shin, H. W., et al. (2016). The KMDS- NATION study: Korean movement disorders society multicenter assessment of non-motor symptoms and quality of life in Parkinson’s disease NATION study group. *Journal of Clinical Neurology (Korea)*, *12*(4), 393–402. <https://doi.org/10.3988/jcn.2016.12.4.393>

Lagrange, E., Krack, P., Moro, E., Ardouin, C., Van Blercom, N., Chabardes, S., et al. (2002). Bilateral subthalamic nucleus stimulation improves health-related quality of life in PD. *Neurology*, *59*(12), 1976-1978. https://doi.org/10.1212/01.wnl.0000037486.82390.1c

Lamichhane, D., Gruber-Baldini, A. L., Reich, S. G., and Shulman, L. M. (2016). Asymmetric responsiveness of disability and health-related quality of life to improvement versus decline in Parkinson’s disease. *Quality of Life Research*, *25*(12), 3139–3145. <https://doi.org/10.1007/s11136-016-1351-1>

Larisch, A., Reuss, A., Oertel, W. H., and Eggert, K. (2011). Does the clinical practice guideline on Parkinson’s disease change health outcomes? A cluster randomized controlled trial. *Journal of Neurology*, *258*(5), 826–834. <https://doi.org/10.1007/s00415-010-5848-1>

Larson, D., Yeh, C., Rafferty, M., and Bega, D. (2022). High satisfaction and improved quality of life with Rock Steady Boxing in Parkinson’s disease: results of a large-scale survey. *Disability and Rehabilitation*, *44*(20), 6034–6041. <https://doi.org/10.1080/09638288.2021.1963854>

Lawrence, B. J., Gasson, N., Kane, R., Bucks, R. S., and Loftus, A. M. (2014). Activities of daily living, depression, and quality of life in Parkinson’s disease. *PLoS ONE*, *9*(7). <https://doi.org/10.1371/journal.pone.0102294>

Lawton, M. P., and Brody, E. M. (1969). Assessment of older people: self-maintaining and instrumental activities of daily living. The gerontologist, 9(3_Part_1), 179-186.
<https://psycnet.apa.org/doi/10.1093/geront/9.3_Part_1.179>

Lawton, M. P., Brody, E., and Médecin, U. (1969). Instrumental activities of daily living (IADL). *The gerontologist*, *9*, 179-186. <https://psycnet.apa.org/doi/10.1037/t06803-000>

Lee, J. H., Choi, M. K., Jung, D., Sohn, Y. H., and Hong, J. Y. (2015). A Structural Model of Health-Related Quality of Life in Parkinson’s Disease Patients. *Western Journal of Nursing Research*, *37*(8), 1062–1080. <https://doi.org/10.1177/0193945914528588>

Lee, K. A., Hicks, G., and Nino-Murcia, G. (1991). Validity and reliability of a scale to assess fatigue. Psychiatry research, 36(3), 291-298. https://doi.org/10.1016/0165-1781(91)90027-M

Leentjens, A. F., Dujardin, K., Pontone, G. M., Starkstein, S. E., Weintraub, D., and Martinez‐Martin, P. (2014). The Parkinson Anxiety Scale (PAS): development and validation of a new anxiety scale. Movement Disorders, 29(8), 1035-1043. <https://doi.org/10.1002/mds.25919>

Leonardi, M., Raggi, A., Pagani, M., Carella, F., Soliveri, P., Albanese, A., et al. (2012). Relationships between disability, quality of life and prevalence of nonmotor symptoms in Parkinson’s disease. *Parkinsonism and Related Disorders*, *18*(1), 35–39. <https://doi.org/10.1016/j.parkreldis.2011.08.011>

Leroi, I., Ahearn, D. J., Andrews, M., Mcdonald, K. R., Byrne, E. J., and Burns, A. (2011). Behavioural disorders, disability and quality of life in Parkinson’s disease. *Age and Ageing*, *40*(5), 614–621. <https://doi.org/10.1093/ageing/afr078>

Lezcano, E., Gómez-Esteban, J. C., Tijero, B., Bilbao, G., Lambarri, I., Rodriguez, O., et al. (2016). Long-term impact on quality of life of subthalamic nucleus stimulation in Parkinson’s disease. *Journal of Neurology*, *263*(5), 895–905.<https://doi.org/10.1007/s00415-016-8077-4>

Li, H., Zhang, M., Chen, L., Zhang, J., Pei, Z., Hu, A., et al. (2010). Nonmotor symptoms are independently associated with impaired health-related quality of life in Chinese patients with Parkinson’s disease. *Movement Disorders*, *25*(16), 2740–2746. <https://doi.org/10.1002/mds.23368>

Li, X. Y., Chen, M. J., Liang, X. N., Yao, R. X., Shen, B., Wu, B., et al. (2023). PDQ-8: A Simplified and Effective Tool Measuring Life Quality in Progressive Supranuclear Palsy. *Journal of Parkinson’s Disease*, *13*(1), 83–91. <https://doi.org/10.3233/JPD-223553>

Liebermann, J. D., Witte, O. W., and Prell, T. (2020). Association between different coping styles and health-related quality of life in people with Parkinson’s disease: a cross-sectional study. *BMJ Open*, *10*(7), e036870. https://doi.org/10.1136/bmjopen-2020- 036870

Liguori, C., De Franco, V., Cerroni, R., Spanetta, M., Mercuri, N. B., Stefani, A., et al. (2021). Sleep problems affect quality of life in Parkinson’s disease along disease progression. *Sleep Medicine*, *81*, 307–311. <https://doi.org/10.1016/j.sleep.2021.02.036>

Linn BS, Linn MW, and Gurel L (1968). Cumulative Illness Rating Scale. J Am Geriatr Soc 1968;16:622–6. <https://doi.org/10.1111/j.1532-5415.1968.tb02103.x>

Livingston, G., Blizard, B., and Mann, A. (1993). Does sleep disturbance predict depression in elderly people? A study in inner London. British Journal of General Practice, 43(376), 445-448.

Lorenzo-García, P., de Arenas-Arroyo, S.N., Cavero-Redondo, I., Guzmán-Pavón, M.J., Priego-Jiménez, S. and Álvarez-Bueno, C., (2023). Physical Exercise Interventions on Quality of Life in Parkinson Disease: A Network Meta-analysis. Journal of Neurologic Physical Therapy, 47(2), .64-74. DOI: 10.1097/NPT.0000000000000414

Lovibond, P. F., and Lovibond, S. H. (1995). The structure of negative emotional states: Comparison of the Depression Anxiety Stress Scales (DASS) with the Beck Depression and Anxiety Inventories. Behaviour Research and Therapy, 33, 335– 343. <https://doi.org/10.1016/0005-7967(94)00075-U>

Lubomski, M., Davis, R. L., and Sue, C. M. (2021). Health-related quality of life for Parkinson’s disease patients and their caregivers. *Journal of movement disorders*, *14*(1), 42. https://doi.org/10.14802%2Fjmd.20079

Luo, N., Low, S., Lau, P. N., Au, W. L., and Tan, L. C. (2009). Is EQ-5D a valid quality of life instrument in patients with Parkinson's disease? A study in Singapore. *Annals of the Academy of Medicine, Singapore*, *38*(6), 521–528. http://dx.doi.org/10.47102/annalsacadmedsg.V38N6p521

Luo, N., Tan, L. C. S., Zhao, Y., Lau, P. N., Au, W. L., and Li, S. C. (2009). Determination of the longitudinal validity and minimally important difference of the 8-item Parkinson’s disease questionnaire (PDQ-8). *Movement Disorders*, *24*(2), 183–187. <https://doi.org/10.1002/mds.22240>

Maeda, T., Shimo, Y., Chiu, S. W., Yamaguchi, T., Kashihara, K., Tsuboi, Y., et al. (2017). Clinical manifestations of nonmotor symptoms in 1021 Japanese Parkinson’s disease patients from 35 medical centers. *Parkinsonism and Related Disorders*, *38*, 54–60. <https://doi.org/10.1016/j.parkreldis.2017.02.024>

Mahoney, F.I. and Barthel, D.W. (1965) ‘Functional Evaluation: The Barthel Index’, Maryland State Medical Journal, 14, pp. 61–65.

Main, C. J. (1983). The modified somatic perception questionnaire (MSPQ). Journal of Psychosomatic Research, 27(6), 503-514. <https://doi.org/10.1016/0022-3999(83)90040-5>

Mantri, S., Chahine, L. M., Nabieva, K., Feldman, R., Althouse, A., Torsney, B., et al. (2022). Demographic Influences on the Relationship Between Fatigue and Quality of Life in Parkinson’s Disease. *Movement Disorders Clinical Practice*, *9*(1), 76–81. <https://doi.org/10.1002/mdc3.13360>

Margolius, A., Cubillos, F., He, Y., Wu, S., Schmidt, P., and Simuni, T. (2018). Predictors of clinically meaningful change in PDQ-39 in Parkinson’s disease. *Parkinsonism andRelated Disorders*, *56*, 93–97. <https://doi.org/10.1016/j.parkreldis.2018.06.034>

Marin, R. S., Biedrzycki, R. C., and Firinciogullari, S. (1991). Reliability and validity of the Apathy Evaluation Scale. *Psychiatry research*, *38*(2), 143–162. <https://doi.org/10.1016/0165-1781(91)90040-v>

Marinus, J., Visser, M., Martı́nez-Martı́n, P., van Hilten, J. J., and Stiggelbout, A. M. (2003). A short psychosocial questionnaire for patients with Parkinson's disease: the SCOPA-PS. Journal of clinical epidemiology, 56(1), 61-67. [https://doi.org/10.1016/S0895-4356(02)00569-3](https://doi.org/10.1016/S0895-%094356(02)00569-3)

Marras, C., McDermott, M. P., Rochon, P. A., Tanner, C. M., Naglie, G., and Lang, A. E. (2008). Predictors of deterioration in health-related quality of life in Parkinson’s disease: Results from the DATATOP trial. *Movement Disorders*, *23*(5), 653–659. <https://doi.org/10.1002/mds.21853>

Martinez Martin, P., Forjaz, M. J., Frades, B., and De Pedro-Cuesta, J. (2005). La rapid assessment disability scale (RAD) enEnfermedad de Parkinson. Gac Sanit, 19(Suppl 1), 68. <http://dx.doi.org/10.13140/RG.2.2.36131.66082>

Martinez-Fernandez, R., Pelissier, P., Quesada, J. L., Klinger, H., Lhommée, E., Schmitt, E., et al. (2016). Postoperative apathy can neutralise benefits in quality of life after subthalamic stimulation for Parkinson’s disease. *Journal of Neurology, Neurosurgery and Psychiatry*, *87*(3), 311–318. https://doi.org/10.1136/jnnp-2014-310189

Martínez-Martín, P., Benito-Lecn, J., Alonso, F., Catalán, M. J., Pondal, M., Tobías, A., et al. (2003). Patient’s, doctors’, and caregivers’ assessment of disability using the UPDRS-ADL section: Are these ratings interchangeable? *Movement Disorders*, *18*(9), 985–992. <https://doi.org/10.1002/mds.10479>

Martínez‐Martín, P., Benito‐León, J., Alonso, F., Catalán, M. J., Pondal, M., and Zamarbide, I. (2004). Health‐related quality of life evaluation by proxy in Parkinson's disease: approach using PDQ‐8 and EuroQoL‐5D. *Movement disorders: official journal of the Movement Disorder Society*, *19*(3), 312-318. <https://doi.org/10.1002/mds.10656>

Martínez‐Martín, P., Benito‐León, J., Alonso, F., Catalán, M. J., Pondal, M., Tobías, A., et al. (2003). Patients', doctors', and caregivers' assessment of disability using the UPDRS‐ADL section: are these ratings interchangeable?.*Movement disorders: official journal of the Movement Disorder Society*, *18*(9), 985-992. https://doi.org/10.1002/mds.10479

Martinez-Martin, P., Deuschl, G., Tonder, L., Schnitzler, A., Houeto, J. L., Timmermann, L., et al. (2020). Interpretation of health-related quality of life outcomes in Parkinson’s disease from the EARLYSTIM Study. *PLoS ONE*, *15*(8 August 2020). https://doi.org/10.1371/journal.pone.0237498

Martínez‐Martín, P., Forjaz, M. J., Cubo, E., Frades, B., De Pedro-Cuesta, J., and ELEP Project Members. (2006). Global versus factor‐related impression of severity in Parkinson's disease: a new clinimetric index (CISI‐PD). *Movement Disorders*, *21*(2), 208-214. <https://doi.org/10.1002/mds.20697>

Martínez-Martín, P., Jiménez-Jiménez, F. J., Carroza García, E., Alonso-Navarro, H., Rubio, L., Calleja, P., et al. (2010). Most of the Quality of Life in Essential Tremor Questionnaire (QUEST) psychometric properties resulted in satisfactory values. *Journal of Clinical Epidemiology*, *63*(7), 767–773. <https://doi.org/10.1016/j.jclinepi.2009.09.001>

Martínez-Martín, P., Payo, B. F., and Grupo Centro for Study of Movement Disorders. (1998). Quality of life in Parkinson’s disease: validation study of the PDQ-39 Spanish version. *Journal of neurology*, *245*, S34-S38. <https://doi.org/10.1007/pl00007737>

Martínez-Martín, P., Rodríguez-Blázquez, C., Forjaz, M. J., Álvarez-Sánchez, M., Arakaki, T., Bergareche-Yarza, A., et al. (2014). Relationship between the MDS-UPDRS domains and the health-related quality of life of Parkinson’s disease patients. *European Journal of Neurology*, *21*(3), 519–524.<https://doi.org/10.1111/ene.12349>

Martinez-Martin, P., Rodriguez-Blazquez, C., Kurtis, M. M., and Chaudhuri, K. R. (2011). The impact of non-motor symptoms on health-related quality of life of patients with Parkinson’s disease. *Movement Disorders*, *26*(3), 399–406. <https://doi.org/10.1002/mds.23462>

Martinez-Martin, P., Rojo-Abuin, J. M., Rizos, A., Rodriguez-Blazquez, C., Trenkwalder, C., Perkins, L., et al. (2017). Distribution and impact on quality of life of the pain modalities assessed by the King’s Parkinson’s disease pain scale. *Npj Parkinson’s Disease*, *3*(1). <https://doi.org/10.1038/s41531-017-0009-1>

Martínez-Martín, P., Serrano-Dueñas, M., and Vaca-Baquero, V. (2005). Psychometric characteristics of the Parkinson’s disease questionnaire (PDQ-39) - Ecuadorian version. *Parkinsonism and Related Disorders*, *11*(5), 297–304. <https://doi.org/10.1016/j.parkreldis.2005.02.003>

Martinez-Martin, P., Serrano-Dueñas, M., Forjaz, M. J., and Serrano, M. S. (2007). Two questionnaires for Parkinson’s disease: Are the PDQ-39 and PDQL equivalent? *Quality of Life Research*, *16*(7), 1221–1230. <https://doi.org/10.1007/s11136-007-9224-2>

McAuliffe, M. J., Baylor, C. R., and Yorkston, K. M. (2017). Variables associated with communicative participation in Parkinson’s disease and its relationship to measures of health-related quality-of-life. *International Journal of Speech-Language Pathology*, *19*(4), 407–417. <https://doi.org/10.1080/17549507.2016.1193900>

Meira, B., Degos, B., Corsetti, E., Doulazmi, M., Berthelot, E., Virbel-Fleischman, C., et al. (2021). Long-term effect of apomorphine infusion in advanced Parkinson’s disease: a real-life study. *Npj Parkinson’s Disease*, *7*(1) <https://doi.org/10.1038/s41531-021-00194-7>

Meissner, W. G., Foubert-Samier, A., Dupouy, S., Debs, R., Gerdelat-Mas, A., Cochen De Cock, V., et al. (2013). Validation of the French version of the MSA health-related Quality of Life scale (MSA-QoL). *Revue Neurologique*, *169*(1), 53–58. <https://doi.org/10.1016/j.neurol.2012.02.011>

Meissner, W. G., Foubert-Samier, A., Dupouy, S., Gerdelat-Mas, A., Debs, R., Marquant, F., et al. (2012). Assessment of quality of life with the multiple system atrophy health-related quality of life scale. *Movement Disorders*, *27*(12), 1574–1577. <https://doi.org/10.1002/mds.25174>

Meng, D., Jin, Z., Chen, K., Yu, X., Wang, Y., Du, W., et al. (2022). Quality of life predicts rehabilitation prognosis in Parkinson’s disease patients: Factors influence rehabilitation prognosis. *Brain and Behavior*, *12*(5). <https://doi.org/10.1002/brb3.2579>

Meng, D., Jin, Z., Gao, L., Wang, Y., Wang, R., Fang, J., et al. (2022). The quality of life in patients with Parkinson’s disease: Focus on gender difference. *Brain and Behavior*, *12*(3). <https://doi.org/10.1002/brb3.2517>

Menon, B., Cherkil, S., Aswathy, S., Unnikrishnan, A. G., and Rajani, G. (2012) The Process and Challenges in the Translation of World Health Organization Quality of Life (WHOQOL- BREF) to a Regional Language; Malayalam. Indian J Psychol Med 2012;34:149-52. <https://doi.org/10.4103%2F0253-7176.101783>

Menon, B., Nayar, R., Kumar, S., Cherkil, S., Venkatachalam, A., Surendran, K., et al. (2015). Parkinson’s disease, depression, and quality-of-life. *Indian Journal of Psychological Medicine*, *37*(2), 144–148. https://doi.org/10.4103/0253-7176.155611

Midlöv, P., Bondesson, Å., Eriksson, T., Petersson, J., Minthon, L., and Höglund, P. (2002). Descriptive study and pharmacotherapeutic intervention in patients with epilepsy or Parkinson’s disease at nursing homes in southern Sweden. *European Journal of Clinical Pharmacology*, *57*(12), 903–910. <https://doi.org/10.1007/s00228-001-0408-3>

Müller, B., Assmus, J., Herlofson, K., Larsen, J. P., and Tysnes, O. B. (2013). Importance of motor vs. non-motor symptoms for health-related quality of life in early Parkinson’s disease. *Parkinsonism and Related Disorders*, *19*(11), 1027–1032. https://doi.org/10.1016/j.parkreldis.2013.07.010

Muthny, F. A. (1989). *Freiburger Fragebogen zur Krankheitsverarbeitung: FKV*. Weinheim: Beltz.

Nakano, T., Kajiyama, Y., Revankar, G.S., Hashimoto, R., Watanabe, Y., Kishima, H., et al. (2021). Neural networks associated with quality of life in patients with Parkinson’s disease. *Parkinsonism and Related Disorders*, 89, 6–12. <https://doi.org/10.1016/j.parkreldis.2021.06.007>

Neumann, N. U., and Schulte, R. M. (1989). Montgomery and Asberg Depression Rating Scale. *Deutsche Fassung. Erlangen: Perimed Fachbuch Verlagsgesellschaft.*

Nicoletti, A., Mostile, G., Stocchi, F., Abbruzzese, G., Ceravolo, R., Cortelli, P., et al. (2017). Factors influencing psychological well-being in patients with Parkinson’s disease. *PLoS ONE*, *12*(12). <https://doi.org/10.1371/journal.pone.0189682>

Nielsen, C., Siersma, V., Ghaziani, E., Beyer, N., Peter Magnusson, S., and Couppé, C. (2020). Health-related quality of life and physical function in individuals with Parkinson’s disease after a multidisciplinary rehabilitation regimen—a prospective cohort feasibility study. *International Journal of Environmental Research and Public Health*, *17*(20), 1–13. <https://doi.org/10.3390/ijerph17207668>

Nojomi, M., Mostafavian, Z., Shahidi, G. A., and Jenkinson, C. (2010). Quality of life in patients with Parkinson’s disease: translation and psychometric evaluation of the Iranian version of PDQ-39. *Journal of research in medical sciences: the official journal of Isfahan University of Medical Sciences*, *15*(2), 63.

Norlin, J. M., Kellerborg, K., Persson, U., Åström, D. O., Hagell, P., Martinez-Martin, P., et al. (2023). Clinical Impression of Severity Index for Parkinson’s Disease and Its Association to Health-Related Quality of Life. *Movement Disorders Clinical Practice*, *10*(3), 392–398. <https://doi.org/10.1002/mdc3.13649>

Nowinski, C. J., Siderowf, A., Simuni, T., Wortman, C., Moy, C., and Cella, D. (2016). Neuro-QoL health-related quality of life measurement system: Validation in Parkinson’s disease. *Movement Disorders*, *31*(5), 725–733. <https://doi.org/10.1002/mds.26546>

Noyes, K., Dick, A. W., and Holloway, R. G. (2006). Pramipexole versus levodopa in patients with early Parkinson’s disease: Effect on generic and disease-specific quality of life. *Value in Health*, *9*(1), 28–38. <https://doi.org/10.1111/j.1524-4733.2006.00078.x>

Nutt, J. G., Siderowf, A. D., Guttman, M., Schmidt, P. N., Zamudio, J. I., Wu, S. S., et al. (2014). Mobility, mood and site of care impact health related quality of life in Parkinson’s disease. *Parkinsonism and Related Disorders*, *20*(3), 274–279 <https://doi.org/10.1016/j.parkreldis.2013.10.004>

Group, T.W., (1998). The World Health Organization quality of life assessment (WHOQOL): development and general psychometric properties. *Social science & medicine*, *46*(12), pp.1569-1585. <https://doi.org/10.1016/S0277-9536(98)00009-4>

Ophey, A., Eggers, C., Dano, R., Timmermann, L., and Kalbe, E. (2018). Health-Related Quality of Life Subdomains in Patients with Parkinson’s Disease: The Role of Gender. *Parkinson’s Disease*, *2018*. <https://doi.org/10.1155/2018/6532320>

Ou, R., Liu, H., Hou, Y., Wei, Q., Cao, B., Zhao, B., et al. (2017). Executive dysfunction, behavioral changes and quality of life in Chinese patients with progressive supranuclear palsy. *Journal of the Neurological Sciences*, *380*, 182–186. <https://doi.org/10.1016/j.jns.2017.07.033>

Ozturk, E. A., Gundogdu, I., Kocer, B., Comoglu, S., and Cakci, A. (2017). Chronic pain in Parkinson’s disease: Frequency, characteristics, independent factors, and relationship with health-related quality of life. *Journal of Back and Musculoskeletal Rehabilitation*, *30*(1), 101–108. <https://doi.org/10.3233/BMR-160720>

Page, M. J., McKenzie, J. E., Bossuyt, P. M., Boutron, I., Hoffmann, T. C., Mulrow, C. D., et al. (2021). The PRISMA 2020 statement: an updated guideline for reporting systematic reviews. *International journal of surgery*, *88*, 105906. https://doi.org/10.1136/bmj.n71

Page, M. J., Moher, D., Bossuyt, P. M., Boutron, I., Hoffmann, T. C., Mulrow, C. D., et al. (2021). PRISMA 2020 explanation and elaboration: updated guidance and exemplars for reporting systematic reviews. *bmj*, *372*. https://doi.org/10.1136/bmj.n160

Palacios, N., Gao, X., Schwarzschild, M., and Ascherio, A. (2012). Declining quality of life in Parkinson disease before and after diagnosis. *Journal of Parkinson’s Disease*, *2*(2), 153–160. <https://doi.org/10.3233/JPD-2012-12083>

Park, H. J., Sohng, K. Y., and Kim, S. (2014). Validation of the Korean version of the 39-Item Parkinson’s disease questionnaire (PDQ-39). *Asian Nursing Research*, *8*(1), 67–74. <https://doi.org/10.1016/j.anr.2014.02.004>

Patton, J. H., Stanford, M. S., and Barratt, E. S. (1995). Factor structure of the Barratt impulsiveness scale. Journal of clinical psychology, 51(6), 768-774. <https://doi.org/10.1002/1097-4679(199511)51:6><768::AID-JCLP2270510607>[3.0.CO](http://3.0.co/);2-1

Perez-Lloret, S., Negre-Pages, L., Damier, P., Delval, A., Derkinderen, P., Destée, A., et al. (2017). L-DOPA-induced dyskinesias, motor fluctuations and health-related quality of life: the COPARK survey. *European Journal of Neurology*, *24*(12), 1532–1538. <https://doi.org/10.1111/ene.13466>

Peto, V., Jenkinson, C., Fitzpatrick, R., and Greenhall, R. (1995). The development and validation of a short measure of functioning and well being for individuals with Parkinson's disease. *Quality of life research: an international journal of quality of life aspects of treatment, care and rehabilitation*, *4*(3), 241–248. <https://doi.org/10.1007/BF02260863>

Pfeiffer E. (1975). A short portable mental status questionnaire for the assessment of organic brain deficit in elderly patients. *Journal of the American Geriatrics Society*, *23*(10), 433–441. <https://doi.org/10.1111/j.1532-5415.1975.tb00927.x>

Picillo, M., Cuoco, S., Amboni, M., Bonifacio, F. P., Bruschi, F., Carotenuto, I., et al. (2019). Validation of the Italian version of the PSP Quality of Life questionnaire. *Neurological Sciences*, *40*(12), 2587–2594. <https://doi.org/10.1007/s10072-019-04010-2>

Powell L.E., and Myers A.M., (1995). The Activities-specific Balance Confidence (ABC) Scale. J Gerontol A Biol Sci Med Sci. 1995 Jan;50A(1):M28-34. <https://doi.org/10.1093/gerona/50A.1.M28>

Prakash, K. M., Nadkarni, N. V., Lye, W. K., Yong, M. H., and Tan, E. K. (2016). The impact of non-motor symptoms on the quality of life of Parkinson’s disease patients: A longitudinal study. *European Journal of Neurology*, *23*(5), 854–860. <https://doi.org/10.1111/ene.12950>

Prell, T., Liebermann, J. D., Mendorf, S., and Zipprich, H. M. (2022). Data on pain coping strategies and their association with quality of life in people with Parkinson’s disease: A cross-sectional study. *Data in Brief*, *42*, 108288. <https://doi.org/10.1016/j.dib.2022.108288>

Prell, T., Liebermann, J. D., Mendorf, S., Lehmann, T., and Zipprich, H. M. (2021). Pain coping strategies and their association with quality of life in people with Parkinson’s disease: A cross-sectional study. *PLoS ONE*, *16*(November). <https://doi.org/10.1371/journal.pone.0257966>

Pusswald, G., Fleck, M., Lehrner, J., Haubenberger, D., Weber, G., and Auff, E. (2012). The “Sense of Coherence” and the coping capacity of patients with Parkinson disease. *International Psychogeriatrics*, *24*(12), 1972–1979. https://doi.org/10.1017/S1041610212001330

Qin, Z., Zhang, L., Sun, F., Fang, X., Meng, C., Tanner, C., et al. (2009). Health related quality of life in early Parkinson’s disease: Impact of motor and non-motor symptoms, results from Chinese levodopa exposed cohort. *Parkinsonism and Related Disorders*,*15*(10), 767–771. <https://doi.org/10.1016/j.parkreldis.2009.05.011>

Qin, Z., Zhang, L., Sun, F., Liu, H., Fang, X., Chan, P., et al. (2009). Depressive symptoms impacting on health-related quality of life in early Parkinson’s disease: Results from Chinese l-dopa exposed cohort. *Clinical Neurology and Neurosurgery*, *111*(9), 733–737. <https://doi.org/10.1016/j.clineuro.2009.07.001>

Quittenbaum, B. H., and Grahn, B. (2004). Quality of life and pain in Parkinson’s disease: A controlled cross-sectional study. *Parkinsonism and Related Disorders*, *10*(3), 129–136. <https://doi.org/10.1016/j.parkreldis.2003.12.001>

Schwarzer R., and Jerusalem M. (1999). SkalenzurErfassung von Lehrer- und Schülermerkmalen [Scales for the Survey of Traits of Teachers and Students], Freie Universität, Berlin,.<http://www.psyc.de/skalendoku.pdf>

Logsdon R.G., Gibbons L.E., McCurry S.M., and Teri L. (1999) Quality of life in Alzheimer's disease: patient and caregiver reports, J. Ment. Health Aging 5 (1) (1999) 21–32.

Radloff, L. S. (1977). The CES-D scale: A self-report depression scale for research in the general population. Applied psychological measurement, 1(3), 385-401. <https://doi.org/10.1177/014662167700100306>

Rafferty, M. R., Schmidt, P. N., Luo, S. T., Li, K., Marras, C., Davis, T. L., et al. (2017). Regular Exercise, Quality of Life, and Mobility in Parkinson’s Disease: A Longitudinal Analysis of National Parkinson Foundation Quality Improvement Initiative Data. *Journal of Parkinson’sDisease*, *7*(1), 193–202. https://doi.org/10.3233/JPD-160912

Raggi, A., Leonardi, M., Covelli, V., Albanese, A., Soliveri, P., Carella, F., et al. (2012). Concordance between severity of disease, prevalence of nonmotor symptoms, patient-reported quality of life and disability and use of medication in Parkinson’s disease. *Neurological Sciences*, *33*(4), 847–853. <https://doi.org/10.1007/s10072-011-0846-3>

Ramadhan, M., and Schrag, A. (2023). The Validity of Health-Related Quality of Life Instruments in Patients With Late-Stage Parkinson’s Disease. *Journal of GeriatricPsychiatry and Neurology*, *36*(3), 225–232. <https://doi.org/10.1177/08919887221119963>

Rascol, O., Negre-Pages, L., Damier, P., Delval, A., Derkinderen, P., Destée, A., et al. (2020). Excessive buccal saliva in patients with Parkinson’s disease of the French COPARK cohort. *Journal of Neural Transmission (Vienna, Austria: 1996)*, *127*(12), 1607–1617. <https://doi.org/10.1007/s00702-020-02249-0>

Rascol, O., Perez-Lloret, S., Damier, P., Delval, A., Derkinderen, P., Destée, A., et al. (2015). Falls in ambulatory non-demented patients with Parkinson’s disease. *Journal of Neural Transmission*, *122*(10), 1447–1455. <https://doi.org/10.1007/s00702-015-1396-2>

Reichmann, H., Boas, J., MacMahon, D., Myllyla, V., Hakala, A., and Reinikainen, K. (2005). Efficacy of combining levodopa with entacapone on quality of life and activities of daily living in patients experiencing wearing-off type fluctuations. *Acta Neurologica Scandinavica*, *111*(1), 21–28. <https://doi.org/10.1111/j.1600-0404.2004.00363.x>

Reilly M.C., Zbrozek A.S., and Dukes E.M. (1993) The validity and reproducibility of a work productivity and activity impairment instrument. Pharmaco economics.;4(5):353-365.<https://doi.org/10.2165/00019053-199304050-00006>

Reisberg B., Borenstein J., Salob S.P., Ferris SH., Franssen E, Georgotas A., (1987) Behavioral symptoms in Alzheimer’s disease: phenomenology and treatment. J Clin Psychiatry.; 48(5,suppl) 9–15.

Resnick, B., and Jenkins, L.S. (2000). Testing the Reliability and Validity of the Self-Efficacy for Exercise Scale. Nursing Research, 49. <http://dx.doi.org/10.1097/00006199-200005000-00007>

Reuther, M., Spottke, E. A., Klotsche, J., Riedel, O., Peter, H., Berger, K., et al. (2007). Assessing health-related quality of life in patients with Parkinson’s disease in a prospective longitudinal study. *Parkinsonism and Related Disorders*, *13*(2), 108–114. <https://doi.org/10.1016/j.parkreldis.2006.07.009>

Richards K. (1987). Techniques for measurement of sleep in critical care. *Focus on critical care*, *14*(4), 34–40.

Ringendahl, H., Werheid, K., Leplow, B., Ellgring, H., Annecke, R., and Emmans, D. (2000). Vorschläge Für Eine Standardisierte psychologische Diagnostik bei Parkinsonpatienten. DerNervenarzt, 71, 946-954. <http://dx.doi.org/10.1007/s001150050691>

Roach, A. J., Frazier, L. P., and Bowden, S. R. (1981). The marital satisfaction scale: Development of a measure for intervention research. Journal of Marriage and the Family, 537-546. <https://doi.org/10.2307/351755>

Rodríguez-Violante, M., Cervantes-Arriaga, A., Corona, T., Martínez-Ramírez, D., Morales-Briceño, H., and Martínez-Martín, P. (2013). Clinical Determinants of Health-related Quality of Life in Mexican Patients with Parkinson’s Disease. *Archives of MedicalResearch*, *44*(2), 110–114. <https://doi.org/10.1016/j.arcmed.2013.01.005>

Roh, J. H., Kim, B. J., Jang, J. H., Seo, W. K., Lee, S. H., Kim, J. H., et al. (2009). The relationship of pain and health-related quality of life in Korean patients with Parkinson’s disease. *Acta Neurologica Scandinavica*, *119*(6), 397–403.<https://doi.org/10.1111/j.1600-0404.2008.01114.x>

Romenets, S. R., Wolfson, C., Galatas, C., Pelletier, A., Altman, R., Wadup, L., et al. (2012). Validation of the non-motor symptoms questionnaire (NMS-Quest). Parkinsonism & related disorders, 18(1), 54-58.<https://doi.org/10.1016/j.parkreldis.2011.08.013>

Rosenberg, M. (1965). Rosenberg self-esteem scale. Journal of Religion and Health. <https://doi.org/10.1037/t01038-000>

Rosenstiel, A. K., and Keefe, F. J. (1983). The use of coping strategies in chronic low back pain patients: relationship to patient characteristics and current adjustment. Pain, 17(1), 33-44. <https://doi.org/10.1016/0304-3959(83)90125-2>

Rosqvist, K., Odin, P., Lorenzl, S., Meissner, W. G., Bloem, B. R., Ferreira, J. J., et al. (2021). Factors Associated with Health-Related Quality of Life in Late-Stage Parkinson’s Disease. *Movement Disorders Clinical Practice*, *8*(4), 563–570. <https://doi.org/10.1002/mdc3.13186>

Rubenstein, L. M., Voelker, M. D., Chrischilles, E. A., Glenn, D. C., Wallace, R. B., and Rodnitzky, R.L. (1998). The usefulness of the functional status questionnaire and medical outcomes study short form in Parkinson's disease research. *Quality of Life Research*, *7*, 279-290. <https://doi.org/10.1023/a:1024973611880>

Rybarczyk, B. (2011). Social and occupational functioning assessment scale (SOFAS). Encyclopedia of clinical neuropsychology, 63, 2313. <https://doi.org/10.1007/978-3-319-57111-9_428>

Ryff C.D. (1989) Happiness is everything, or is it? Explorations on the meaning of psychological well-being. J Pers SocPsychol.: 57:1069–1081.<https://psycnet.apa.org/doi/10.1037/0022-3514.57.6.1069>

Ryff, C. D. and Keyes, C. L. M. (1995). The structure of psychological well-being. Journal of Personality and Social Psychology, 69, 719–727 <https://psycnet.apa.org/doi/10.1037/0022-3514.69.4.719>

Saeedian, R. G., Nagyova, I., Klein, D., Skorvanek, M., Rosenberger, J., Gdovinova, Z., et al. (2014). Self-rated health mediates the association between functional status and health-related quality of life in Parkinson’s disease. *Journal of Clinical Nursing*, *23*(13–14), 1970–1977. <https://doi.org/10.1111/jocn.12442>

Sanchez-Luengos, I., Lucas-Jiménez, O., Ojeda, N., Peña, J., Gómez-Esteban, J. C., Gómez-Beldarrain, et al. (2022). Predictors of health-related quality of life in Parkinson’s disease: the impact of overlap between health-related quality of life and clinical measures. *Quality of Life Research*, *31*(11), 3241–3252. <https://doi.org/10.1007/s11136-022-03187-y>

Santangelo, G., Barone, P., Cuoco, S., Raimo, S., Pezzella, D., Picillo, M., et al. (2014). Apathy in untreated, de novo patients with Parkinson's disease: validation study of Apathy Evaluation Scale. *Journal of neurology*, *261*(12), 2319–2328. <https://doi.org/10.1007/s00415-014-7498-1>

Santos-García, D., and De La Fuente-Fernández, R. (2013). Impact of non-motor symptoms on health-related and perceived quality of life in Parkinson’s disease. *Journal of the Neurological Sciences*, *332*(1–2), 136–140. <https://doi.org/10.1016/j.jns.2013.07.005>

Sarason I.G., Levine H.M., Basham R.B., and Sarason B. R. (1983). Assessing social support: the social support questionnaire. J Pers Soc Psychol; 44:127–139<https://psycnet.apa.org/doi/10.1037/0022-3514.44.1.127>

Savci, C., and Sendir, M. (2009). Evaluation of health related quality of life in patients with Parkinsons disease. *Neurosciences (Riyadh, Saudi Arabia)*, *14*(1), 60–66.

Scheier, M. F., and Carver, C. S. (1985). Optimism, coping, and health: assessment and implications of generalized outcome expectancies. Health psychology, 4(3), 219. <https://doi.org/10.1037/0278-6133.4.3.219>

Schmidt J., Lamprecht F., and Wittmann W. W. (1989). Satisfaction with inpatient management. Development of a questionnaire and initial validity studies. PsychotherPsychosomMedPsychol 39:248–255

Schönenberg, A., Zipprich, H.M., Teschner, U., Grosskreutz, J., Witte, O.W. and Prell, T., (2021). Impact of subthreshold depression on health-related quality of life in patients with Parkinson’s disease based on cognitive status*. Health and quality of life outcomes, 19,* pp.1-8 <https://doi.org/10.1186/s12955-021-01753-5>

Schrag, A., Geser, F., Stampfer Kountchev, M., Seppi, K., Sawires, M., Köllensperger, M., et al. (2006). Health-related quality of life in multiple system atrophy. *Movement Disorders*, *21*(6), 809–815. <https://doi.org/10.1002/mds.20808>

Schrag, A., Selai, C., Quinn, N. and Hobart, J. (2005). Measuring health-related quality of life in patients with progressive supranuclear palsy. *Neurocase*, *11*(4), pp.246-249. <https://doi.org/10.1080/13554790590963068>

Schrag, A., Hovris, A., Morley, D., Quinn, N., and Jahanshahi, M. (2003). Young- versus older-onset Parkinson’s disease: Impact of disease and psychosocial consequences. *Movement Disorders*, *18*(11), 1250–1256. <https://doi.org/10.1002/mds.10527>

Schrag, A., Jenkinson, C., Selai, C., Mathias, C., and Quinn, N. (2007). Testing the validity of the PDQ-39 in patients with MSA. *Parkinsonism and Related Disorders*, *13*(3), 152–156. <https://doi.org/10.1016/j.parkreldis.2006.08.008>

Schrag, A., Selai, C., Mathias, C., Low, P., Hobart, J., Brady, N., et al. (2007). Measuring health-related quality of life in MSA: the MSA-QoL. *Movement disorders: official journal of the Movement Disorder Society*, *22*(16), 2332–2338. <https://doi.org/10.1002/mds.21649>

Schrag, A., Selai, C., Quinn, N., Lees, A., Litvan, I., Lang, A., et al. (2006). Measuring quality of life in PSP: the PSP-QoL. *Neurology*, *67*(1), 39–44. <https://doi.org/10.1212/01.wnl.0000223826.84080.97>

Schwab, R. S., and England A. C. J. (1969) Projection technique for evaluating surgery in Parkinson's disease. In: Gillingham FJ, Donaldson IML, editors. Third Symposium on Parkinson's Disease. Edinburgh, Scotland: E & S Livingstone,.152-157.

Serrano-Dueñas, M., Martínez-Martín, P., and Vaca-Baquero, V. (2004). Validation and cross-cultural adjustment of PDQL-questionnaire, Spanish version (Ecuador) (PDQL-EV). *Parkinsonism and Related Disorders*, *10*(7), 433–437. https://doi.org/10.1016/j.parkreldis.2004.05.002

Seymour, K. C., Pickering, R., Rochester, L., Roberts, H. C., Ballinger, C., Hulbert, S., et al. (2019). Multicentre, randomised controlled trial of PDSAFE, a physiotherapist-delivered fall prevention programme for people with Parkinson’s. *Journal of Neurology, Neurosurgery & Psychiatry*, *90*(7), 774-782. [http s://doi.org/10.1136/jnnp-2018-319448](https://doi.org/10.1136/jnnp-2018-319448)

Shafazand, S., Wallace, D. M., Arheart, K. L., Vargas, S., Luca, C. C., Moore, H., et al. (2017). Insomnia, sleep quality, and quality of life in mild to moderate parkinson’s disease. *Annals of the American Thoracic Society*, *14*(3), 412–419. <https://doi.org/10.1513/AnnalsATS.201608-625OC>

Shah-Zamora, D., Allen, A. M., Rardin, L., Ivancic, M., Durham, K., Hickey, P., et al. (2021). Mindfulness based stress reduction in people with Parkinson’s disease and their care partners. *Complementary Therapies in Clinical Practice*, *43*. <https://doi.org/10.1016/j.ctcp.2021.101377>

Shalash, A. S., Hamid, E., Elrassas, H. H., Bedair, A. S., Abushouk, A. I., Khamis, M., et al. (2018). Non-motor symptoms as predictors of quality of life in Egyptian patients with Parkinson’s disease: A cross-sectional study using a culturally adapted 39-item Parkinson’s disease questionnaire. *Frontiers in Neurology*, *9*(MAY). <https://doi.org/10.3389/fneur.2018.00357>

Shearer, J., Green, C., Counsell, C. E., and Zajicek, J. P. (2012). The impact of motor and non motor symptoms on health state values in newly diagnosed idiopathic Parkinson’s disease. *Journal of Neurology*, *259*(3), 462–468. <https://doi.org/10.1007/s00415-011-6202-y>

Shimbo, T., Goto, M., Morimoto, T., Hira, K., Takemura, M., Matsui, K., Yoshida, A. and Fukui, T. (2004). Association between patient education and health-related quality of life in patients with Parkinson's disease. *Quality of Life Research*, *13*, pp.81-89. <https://doi.org/10.1023/b:qure.0000015306.59840.95>

Shimo, Y., Maeda, T., Chiu, S. W., Yamaguchi, T., Kashihara, K., Tsuboi, Y., et al. (2021). Influence of istradefylline on non-motor symptoms of Parkinson’s disease: A subanalysis of a 1-year observational study in Japan (J-FIRST). *Parkinsonism and Related Disorders*, *91*, 115–120. <https://doi.org/10.1016/j.parkreldis.2021.09.015>

Schönenberg, A., Santos García, D., Mir, P., Wu, J. J., Heimrich, K. G., Mühlhammer, H. M. et al. (2023). Using network analysis to explore the validity and influential items of the Parkinson’s Disease Questionnaire-39. *Scientific Reports*, *13*(1)
https://doi.org/10.1038/s41598-023-34412-4.

Siderowf, A., Ravina, B., and Glick, H. A. (2002). Preference-based quality-of-life in patients with Parkinson’s disease. *Neurology*, *59*(1), 103-108. <https://doi.org/10.1212/WNL.59.1.103>

Simpson, J., Eccles, F. and Zarotti, N. (2021). Extended evidence-based guidance on psychological interventions for psychological difficulties in individuals with Huntington’s Disease. *Parkinson’s Disease, Motor Neurone Disease, and Multiple Sclerosis*. <https://doi.org/10.5281/zenodo.4593883>

Simpson, J., Lekwuwa, G., and Crawford, T. (2014). Predictors of quality of life in people with Parkinson’s disease: Evidence for both domain specific and general relationships. *Disability and Rehabilitation*, *36*(23), 1964–1970. <https://doi.org/10.3109/09638288.2014.883442>

Sintonen, H. (2001). The 15D instrument of health-related quality of life: properties and applications. *Annals of medicine*, *33*(5), 328–336. <https://doi.org/10.3109/07853890109002086>

Sitzia, J., Haddrell, V., and Rice-Oxley, M. (1998). Evaluation of a nurse-led multidisciplinary neurological rehabilitation programme using the Nottingham Health Profile. *Clinical rehabilitation*, *12*(5), 389-394. <https://doi.org/10.1191/026921598675167321>

Skorvanek, M., Martinez-Martin, P., Kovacs, N., Zezula, I., Rodriguez-Violante, M., Corvol, J. C., et al. (2018). Relationship between the MDS-UPDRS and Quality of Life: A large multicenter study of 3206 patients. *Parkinsonism and Related Disorders*, *52*, 83–89. <https://doi.org/10.1016/j.parkreldis.2018.03.027>

Smeets, R. M. W., and Dingemans, P. M. A. J. (1993). Composite international diagnostic interview (CIDI), Version 1.1. World Health Organization, Amsterdam/Geneva.

Snaith, R. P., Bridge, G. W. K., and Hamilton, M. (1976). The Leeds scales for the self-assessment of anxiety and depression. The British Journal of Psychiatry, 128(2), 156-165. <https://doi.org/10.1192/bjp.128.2.156>

Sockeel, P., Dujardin, K., Devos, D., Denève, C., Destée, A., and Defebvre, L. (2006). The Lille apathy rating scale (LARS), a new instrument for detecting and quantifying apathy: validation in Parkinson’s disease. *Journal of Neurology, Neurosurgery&Psychiatry*, *77*(5), 579-584. <https://doi.org/10.1136%2Fjnnp.2005.075929>

Soh, S. E., McGinley, J. L., Watts, J. J., Iansek, R., Murphy, A. T., Menz, H. B., et al. (2013). Determinants of health-related quality of life in people with Parkinson’s disease: a path analysis. *Quality of life research*, *22*, 1543-1553. https://doi.org/10.1007/s11136-012-0289-1

Soh, S. E., McGinley, J. L., Watts, J., Iansek, R., and Morris, M. E. (2012). Rural living and health-related quality of life in Australians with Parkinson's disease. <http://dx.doi.org/10.22605/RRH2158>

Soh, S. E., Morris, M. E., Watts, J. J., Mcginley, J. L., and Iansek, R. (2016). Health-related quality of life in people with Parkinson’s disease receiving comprehensive care. *Australian Health Review*, *40*(6), 613–618. <https://doi.org/10.1071/AH15113>

Soldatos, C. R., Dikeos, D. G., and Paparrigopoulos, T. J. (2000). Athens Insomnia Scale: validation of an instrument based on ICD-10 criteria. *Journal of psychosomatic research*, *48*(6), 555–560. <https://doi.org/10.1016/s0022-3999(00)00095-7>

Song, W., Guo, X., Chen, K., Chen, X., Cao, B., Wei, Q., et al. (2014). The impact of non-motor symptoms on the Health-Related Quality of Life of Parkinson’s disease patients from Southwest China. *Parkinsonism and Related Disorders*, *20*(2), 149–152. <https://doi.org/10.1016/j.parkreldis.2013.10.005>

Soulas, T., Storme, M., Martínez-Martín, P., Pichlak, M., Gurruchaga, J. M., Palfi, S., et al. (2016). Assessing health-related quality of life with the SCOPA-PS in French individuals with Parkinson’s disease having undergone DBS-STN: A validation study. *Revue Neurologique*, *172*(4–5), 281–288 <https://doi.org/10.1016/j.neurol.2015.10.010>

Soulas, T., Storme, M., Martínez-Martín, P., Pichlak, M., Gurruchaga, J. M., Palfi, S., et al. (2016). Assessing health-related quality of life with the SCOPA-PS in French individuals with Parkinson's disease having undergone DBS-STN: A validation study. RevueNeurologique, 172(4-5), 281-288. <https://doi.org/10.1016/j.neurol.2015.10.010>

Spadaro, L., Bonanno, L., Di Lorenzo, G., Bramanti, P., and Marino, S. (2013). Health-related quality of life in Parkinson’s disease patients in northeastern Sicily, Italy: An ecological perspective. *Neural Regeneration Research*, *8*(17), 1615–1622. <https://doi.org/10.3969/j.issn.1673-5374.2013.17.010>

Spielberger, C., Gorsuch, R. and Lushene, R. (1970). Manual for the State Trait Anxiety Inventory. Palo Alto, California, Consulting Psychologist Press

Spliethoff-Kamminga, N. G., Zwinderman, A. H., Springer, M. P., and Roos, R. A. (2003). Psychosocial problems in Parkinson's disease: evaluation of a disease-specific questionnaire. *Movement disorders: official journal of the Movement Disorder Society*, *18*(5), 503–509 <https://doi.org/10.1002/mds.10388>

Starkstein, S. E., Mayberg, H. S., Preziosi, T. J., Andrezejewski, P., Leiguarda, R., and Robinson, R. G. (1992). Reliability, validity, and clinical correlates of apathy in Parkinson's disease. *The Journal of neuropsychiatry and clinical neurosciences*, *4*(2), 134–139. <https://doi.org/10.1176/jnp.4.2.134>

Starkstein, S. E., Petracca, G., Chemerinski, E., and Kremer, J. (2001). Syndromic validity of apathy in Alzheimer's disease. *The American journal of psychiatry*, *158*(6), 872–877. <https://doi.org/10.1176/appi.ajp.158.6.872>

Stiasny-Kolster, K., Mayer, G., Schäfer, S., Möller, J. C., Heinzel-Gutenbrunner, M., and Oertel, W. H. (2007). The REM sleep behavior disorder screening questionnaire--a new diagnostic instrument. *Movement disorders : official journal of the Movement Disorder Society*, *22*(16), 2386–2393. <https://doi.org/10.1002/mds.21740>

Stocchi, F., Radicati, F. G., Chaudhuri, K. R., Johansson, A., Padmakumar, C., Falup-Pecurariu, C., et al. (2018). The Parkinson's Disease Composite Scale: results of the first validation study. *European journal of neurology*, *25*(3), 503–511. <https://doi.org/10.1111/ene.13529>

Suarez, G. A., Opfer-Gehrking, T. L., Offord, K. P., Atkinson, E. J., O'Brien, P. C., and Low, P. A. (1999). The Autonomic Symptom Profile: a new instrument to assess autonomic symptoms. *Neurology*, *52*(3), 523–528. <https://doi.org/10.1212/wnl.52.3.523>

Suzukamo, Y., Ohbu, S., Kondo, T., Kohmoto, J., and Fukuhara, S. (2006). Psychological adjustment has a greater effect on health-related quality of life than on severity of disease in Parkinson’s disease. *Movement Disorders*, *21*(6), 761–766. https://doi.org/10.1002/mds.20817

Tan, L. C. S., Luo, N., Nazri, M., Li, S. C., and Thumboo, J. (2004). Validity and reliability of the PDQ-39 and the PDQ-8 in English-speaking Parkinson’s disease patients in Singapore. *Parkinsonism and Related Disorders*, *10*(8), 493–499. <https://doi.org/10.1016/j.parkreldis.2004.05.007>

Terrens, A. F., Soh, S. E., and Morgan, P. (2021). Perceptions of aquatic physiotherapy and health-related quality of life among people with Parkinson’s disease. *Health Expectations*, *24*(2), 566–577. <https://doi.org/10.1111/hex.13202>

Terriff, D. L., Williams, J. V. A., Patten, S. B., Lavorato, D. H., and Bulloch, A. G. M. (2012). Patterns of disability, care needs, and quality of life of people with Parkinson’s disease in a general population sample. *Parkinsonism and Related Disorders*, *18*(7), 828–832. <https://doi.org/10.1016/j.parkreldis.2012.03.026>

Thach, A., Jones, E., Pappert, E., Pike, J., Wright, J., and Gillespie, A. (2021). Real-world assessment of the impact of “OFF” episodes on health-related quality of life among patients with Parkinson’s disease in the United States. *BMC Neurology*, *21*(1). <https://doi.org/10.1186/s12883-021-02074-2>

Whoqol Group, (1998). Development of the World Health Organization WHOQOL-BREF quality of life assessment. *Psychological medicine*, *28*(3), pp.551-558.

Tickle-Degnen, L., Ellis, T., Saint-Hilaire, M. H., Thomas, C. A., and Wagenaar, R. C. (2010). Self-management rehabilitation and health-related quality of life in Parkinson’s disease: A randomized controlled trial. *Movement Disorders*, *25*(2), 194–204. <https://doi.org/10.1002/mds.22940>

Tinetti, M. E., Richman, D., and Powell, L. (1990). Falls efficacy as a measure of fear of falling. Journal of gerontology, 45(6), 239-243. <https://doi.org/10.1093/geronj/45.6.P239>

Tolosa, E., Ebersbach, G., Ferreira, J. J., Rascol, O., Antonini, A., Foltynie, T., et al. (2021). The Parkinson’s Real-World Impact Assessment (PRISM) Study: A European Survey of the Burden of Parkinson’s Disease in Patients and their Carers. In *Journal of Parkinson’s Disease* (Vol. 11, Issue 3, pp. 309–1323). IOS Press BV. <https://doi.org/10.3233/JPD-212611>

Tran, T. N., Ha, U. N. Le, Nguyen, T. M., Nguyen, T. D., Vo, K. N. C., et al. (2021). The effect of Non-Motor symptoms on Health-Related quality of life in patients with young onset Parkinson’s Disease: A single center Vietnamese Cross-Sectional study. *Clinical Parkinsonism and Related Disorders*, *5*. <https://doi.org/10.1016/j.prdoa.2021.100118>

Trang, I., Katz, M., Galifianakis, N., Fairclough, D., Sillau, S. H., Miyasaki, J., et al. (2020). Predictors of general and health-related quality of life in Parkinson’s disease and related disorders including caregiver perspectives. *Parkinsonism and Related Disorders*, *77*, 5–10. <https://doi.org/10.1016/j.parkreldis.2020.05.036>

Tröster, A. I., Pahwa, R., Fields, J. A., Tanner, C. M., and Lyons, K. E. (2005). Quality of life in Essential Tremor Questionnaire (QUEST): development and initial validation. Parkinsonism & related disorders, 11(6), 367-373. <https://doi.org/10.1016/j.parkreldis.2005.05.009>

Tsuboi, T., Lopes, J. L. M. L. J., Moore, K., Patel, B., Legacy, J., Ratajska, A. M., et al. (2021). Long-term clinical outcomes of bilateral GPi deep brain stimulation in advanced Parkinson’s disease: 5 years and beyond. In *Journal of Neurosurgery* (Vol. 135, Issue 2, pp.601–610). American Association of Neurological Surgeons. <https://doi.org/10.3171/2020.6.JNS20617>

Tsuru, A., Matsui, K., Kimura, A., Yoshiike, T., Otsuki, R., Nagao, K., et al. (2022). Sleep disturbance and health-related quality of life in Parkinson’s disease: A clear correlation between health-related quality of life and subjective sleep quality. *Parkinsonism and Related Disorders*, *98*, 86–91. <https://doi.org/10.1016/j.parkreldis.2022.04.014>

Tu, X. J., Hwang, W. J., Hsu, S. P., and Ma, H. I. (2017). Responsiveness of the short-form health survey and the Parkinson’s disease questionnaire in patients with Parkinson’s disease. *Health and Quality of Life Outcomes*, *15*(1). <https://doi.org/10.1186/s12955-017-0642-8>

Tu, X. J., Hwang, W. J., Ma, H. I., Chang, L. H., and Hsu, S. P. (2017). Determinants of generic and specific health-related quality of life in patients with Parkinson’s disease. *PLoS ONE*, *12*(6). <https://doi.org/10.1371/journal.pone.0178896>

Ueno, T., Kon, T., Haga, R., Nishijima, H., Arai, A., and Tomiyama, M. (2020). *Assessing the relationship between non-motor symptoms and health-related quality of life in Parkinson’s disease: a retrospective observational cohort study*. <https://doi.org/10.1007/s10072-020-04406-5/Published>

Urell, C., Zetterberg, L., Hellström, K., and Anens, E. (2021). Factors explaining physical activity level in Parkinson's disease: A gender focus. *Physiotherapy Theory and Practice*, *37*(4), 507–516. <https://doi.org/10.1080/09593985.2019.1630875>

Ustün, T. B., Chatterji, S., Kostanjsek, N., Rehm, J., Kennedy, C., Epping-Jordan, J., et al. (2010). Developing the World Health Organization Disability Assessment Schedule 2.0. *Bulletin of the World Health Organization*, *88*(11), 815–823. <https://doi.org/10.2471/BLT.09.067231>

Valeikiene, V., Ceremnych, J., Alekna, V., and Juozulynas, A. (2008). Differences in WHOQOL-100 domain scores in Parkinson's disease and osteoarthritis. *Medical science monitor: international medical journal of experimental and clinical research*, *14*(4), CR221–CR227.

Valkovic, P., Harsany, J., Hanakova, M., Martinkova, J., and Benetin, J. (2014). Nonmotor Symptoms in Early- and Advanced-Stage Parkinson’s Disease Patients onDopaminergic Therapy: How Do They Correlate with Quality of Life? *ISRN Neurology*, *2014*, 1–4. <https://doi.org/10.1155/2014/587302>

van der Eijk, M., Faber, M. J., Ummels, I., Aarts, J. W., Munneke, M., and Bloem, B. R. (2012). Patient-centeredness in PD care: development and validation of a patient experience questionnaire. *Parkinsonism & related disorders*, *18*(9), 1011–1016. <https://doi.org/10.1016/j.parkreldis.2012.05.017>

Virués-Ortega, J., Carod-Artal, F. J., Serrano-Dueñas, M., Ruiz-Galeano, G., Meza-Rojas, G., Velázquez, C., et al. (2009). Cross-cultural validation of the Scales for Outcomes in Parkinson's Disease-Psychosocial questionnaire (SCOPA-PS) in four Latin American countries. *Value in Health*, *12*(2), 385-391. https://doi.org/10.1111/j.15244733.2008.00436.x

Visser, M., Marinus, J., Stiggelbout, A. M., and van Hilten, J. J. (2004). Assessment of autonomic dysfunction in Parkinson's disease: the SCOPA‐AUT. Movement disorders: official journal of the Movement Disorder Society, 19(11), 1306-1312.<https://doi.org/10.1002/mds.20153>

Visser, M., van Rooden, S.M., Verbaan, D., Marinus, J., Stiggelbout, A.M. and van Hilten, J.J., (2008). A comprehensive model of health-related quality of life in Parkinson’s disease. *Journal of neurology*, *255*, pp.1580-1587. <https://doi.org/10.1007/s00415-008-0994-4>

Visser, M., Verbaan, D., Van Rooden, S., Marinus, J., Van Hilten, J., and Stiggelbout, A. (2009). A longitudinal evaluation of health-related quality of life of patients with Parkinson’s disease. *Value in Health*, *12*(2), 392–396. <https://doi.org/10.1111/j.1524-4733.2008.00430.x>

Ware Jr, J. E., Kosinski, M., and Keller, S. D. (1996). A 12-Item Short-Form Health Survey: construction of scales and preliminary tests of reliability and validity. *Medical care*, 220-233. <https://doi.org/10.1097/00005650-199603000-00003>

Ware, J. E., Jr, and Sherbourne, C. D. (1992). The MOS 36-item short-form health survey (SF-36). I. Conceptual framework and item selection. *Medical care*, *30*(6), 473–483. <https://psycnet.apa.org/doi/10.1097/00005650-199206000-00002>

Ware, J., Jr, Kosinski, M., and Keller, S. D. (1996). A 12-Item Short-Form Health Survey: construction of scales and preliminary tests of reliability and validity. *Medical care*, *34*(3), 220–233. <https://doi.org/10.1097/00005650-199603000-00003>

Watanabe, H., Saiki, H., Chiu, S. W., Yamaguchi, T., Kashihara, K., Tsuboi, Y., et al. (2020). Real-World Nonmotor Changes in Patients with Parkinson’s Disease and Motor Fluctuations: J-FIRST. *Movement Disorders Clinical Practice*, *7*(4), 431–439. <https://doi.org/10.1002/mdc3.12939>

Watson, D., Clark, L. A., and Tellegen, A. (1988). Development and validation of brief measures of positive and negative affect: the PANAS scales. *Journal of personality and social psychology*, *54*(6), 1063. <https://psycnet.apa.org/doi/10.1037/0022-3514.54.6.1063>

Webster, K., Odom, L., Peterman, A., Lent, L. and Cella, D., (1999). The Functional Assessment of Chronic Illness Therapy (FACIT) measurement system: validation of version 4 of the core questionnaire. *Quality of Life Research*, pp.604-604.

Weintraub, D., Hoops, S., Shea, J. A., Lyons, K. E., Pahwa, R., Driver-Dunckley, E. D., et al. (2009). Validation of the questionnaire for impulsive-compulsive disorders in Parkinson’s disease. Movement Disorders, 24(10), 1461–1467. <https://doi.org/10.1002/mds.22571>

Weitkunat, R., Letzel, H., Kanowski, S., and Grobe-Einsler, R. (1993). Clinical and psychometric evaluation of the efficacy of nootropic drugs: Characteristics of several procedures. Zeitschrift für Gerontopsychologie und-psychiatrie.

Welsh, M., McDermott, M. P., Holloway, R. G., Plumb, S., Pfeiffer, R., Hubble, J., et al. (2003). Development and testing of the Parkinson's disease quality of life scale. *Movement disorders : official journal of the Movement Disorder Society*, *18*(6), 637–645. <https://doi.org/10.1002/mds.10424>

Wenning GK, Tison F, Seppi K, et al. (2004). Development and validation of the Unified Multiple System Atrophy Rating Scale (UMSARS). *Mov Disord*;19:1391–1402<https://doi.org/10.1002/mds.20255>

Whitworth, S. R., Loftus, A. M., Skinner, T. C., Gasson, N., Barker, R. A., Bucks, R. S., et al. (2013). Personality affects aspects of health-related quality of life in Parkinson's disease via psychological coping strategies. *Journal of Parkinson's disease*, *3*(1), 45-53. <https://doi.org/10.3233/JPD-120149>

Winter, Y., von Campenhausen, S., Arend, M., Longo, K., Boetzel, K., Eggert, K., et al. (2011). Health-related quality of life in multiple system atrophy and progressive supranuclear palsy. *Neurodegenerative Diseases*, *8*(6), 438–446. <https://doi.org/10.1159/000325829>

Winter, Y., von Campenhausen, S., Arend, M., Longo, K., Boetzel, K., Eggert, K., et al. (2011). Health-related quality of life and its determinants in Parkinson’s disease: Results of an Italian cohort study. *Parkinsonism and Related Disorders*, *17*(4), 265–269. <https://doi.org/10.1016/j.parkreldis.2011.01.003>

Winter, Y., von Campenhausen, S., Gasser, J., Seppi, K., Reese, J. P., Pfeiffer, K. P., et al. (2010). Social and clinical determinants of quality of life in Parkinson’s disease in Austria: A cohort study. *Journal of Neurology*, *257*(4), 638–645. <https://doi.org/10.1007/s00415-009-5389-7>

Winter, Y., von Campenhausen, S., Popov, G., Reese, J. P., Balzer-Geldsetzer, M., Kukshina, A., et al. (2010). Social and clinical determinants of quality of life in Parkinson’s disease in a Russian cohort study. *Parkinsonism and Related Disorders*, *16*(4), 243–248. <https://doi.org/10.1016/j.parkreldis.2009.11.012>

World Health Organization. (1999). The World Health Organization disability assessment schedule phase II field trial instrument. The World Health Organization, Geneva, Switzerland.

Wu, P. L., Lee, M., Wu, S. L., Ho, H. H., Chang, M. H., Lin, H. S., et al. (2021). Effects of home-based exercise on motor, non-motor symptoms and health-related quality of life in Parkinsonʼs disease patients: A randomized controlled trial. *Japan Journal of Nursing Science*, *18*(3). <https://doi.org/10.1111/jjns.12418>

Xiao, Y., Zhang, L., Wei, Q., Ou, R., Hou, Y., Liu, K., et al. (2022). Health-related quality of life in patients with multiple system atrophy using the EQ- 5D-5L. *Brain and Behavior*, *12*(10). <https://doi.org/10.1002/brb3.2774>

Yamabe, K., Liebert, R., Flores, N., and Pashos, C. (2018). Health-related quality-of-life, work productivity, and economic burden among patients with Parkinson’s disease in Japan. *Journal of Medical Economics*, *21*(12), 1206–1212. <https://doi.org/10.1080/13696998.2018.1522638>

Yesavage, J. A., Brink, T. L., Rose, T. L., Lum, O., Huang, V., Adey, M., et al. (1982). Development and validation of a geriatric depression screening scale: a preliminary report. Journal of psychiatric research, 17(1), 37-49. <https://doi.org/10.1016/0022-3956(82)90033-4>

Yoo, S. W., Kim, J. S., Oh, Y. S., Ryu, D. W., and Lee, K. S. (2019). Excessive daytime sleepiness and its impact on quality of life in de novo Parkinson’s disease. *Neurological Sciences*, *40*(6), 1151–1156. <https://doi.org/10.1007/s10072-019-03785-8>

Yoon, J. E., Kim, J. S., Jang, W., Park, J., Oh, E., Youn, J., et al. (2017). Gender Differences of Nonmotor Symptoms Affecting Quality of Life in Parkinson Disease. *Neurodegenerative Diseases*, *17*(6), 276–280. <https://doi.org/10.1159/000479111>

Zahra, M., Durand-Zaleski, I., Górecki, M., WalleserAutiero, S., Barnett, G., and Schüpbach, W. M. M. (2020). Parkinson’s disease with early motor complications: Predicting EQ-5D-3L utilities from PDQ-39 data in the EARLYSTIM trial. *Health and Quality of Life Outcomes*, *18*(1). <https://doi.org/10.1186/s12955-020-01299-y>

Zarotti, N., Eccles, F.J., Foley, J.A., Paget, A., Gunn, S., Leroi, I. et al. ( 2021). Psychological interventions for people with Parkinson’s disease in the early 2020s: Where do we stand?. *Psychology and Psychotherapy: Theory, Research and Practice*, *94*(3), 760-797. <https://doi.org/10.1111/papt.12321>

Zhang, J. L., and Chan, P. (2012). Reliability and validity of PDQ-39: A quality-of-life measure for patients with PD in China. *Quality of Life Research*, *21*(7), 1217–1221. <https://doi.org/10.1007/s11136-011-0026-1>

Zhao, Y. J., Tan, L. C. S., Lau, P. N., Au, W. L., Li, S. C., and Luo, N. (2008). Factors affecting health-related quality of life amongst Asian patients with Parkinson’s disease. *European Journal of Neurology*, *15*(7), 737–742. <https://doi.org/10.1111/j.1468-1331.2008.02178.x>

Zigmond, A. S., and Snaith, R. P. (1983). The hospital anxiety and depression scale. Acta psychiatrica scandinavica, 67(6), 361-370. <https://doi.org/10.1111/j.1600-0447.1983.tb09716.x>

Zimmerman, G. J., D’Antonio, L. L., Iacono, R. P., and Scerrati, M. (2004). Health related quality of life in patients with Parkinson’s disease two years following posteroventral pallidotomy. *Acta Neurochirurgica*, *146*(12), 1293–1299. <https://doi.org/10.1007/s00701-004-0385-2>

Zipprich, H.M., Mendorf, S., Schönenberg, A. and Prell, T. (2021). Self‐reported nonadherence to medication is not associated with health‐related quality of life in parkinson’s disease. *Brain Sciences*, *11*(2), 1–8. <https://doi.org/10.3390/brainsci11020273>

Zipprich, H. M., Mendorf, S., Schönenberg, A., and Prell, T. (2022). The impact of poor medication knowledge on health-related quality of life in people with Parkinson’s disease: a mediation analysis. *Quality of Life Research*, *31*(5), 1473–1482. <https://doi.org/10.1007/s11136-021-03024-8>

Žiropada, L., Stefanova, E., Potrebić, A., and Kostić, V. S. (2009). Quality of life in Serbian patients with Parkinson’s disease. *Quality of Life Research*, *18*(7), 833–839. <https://doi.org/10.1007/s11136-009-9500-4>

Zung, W. W. (1965) A self-rating depression scale. Arch Gen Psychiatry;12: 63–70 <https://doi.org/10.1001/archpsyc.1965.01720310065008>

Zung, W. W. (1972). The Depression Status Inventory: an adjunct to the Self-Rating Depression Scale. *Journal of clinical psychology, 28 4*, 539-43. <https://doi.org/10.1002/1097-4679(197210)28:4%3C539::aid-jclp2270280427%3E3.0.co;2-s>
